# Integrin-specific hydrogels modulate transplanted human bone marrow-derived mesenchymal stem cell survival, engraftment, and reparative activities

**DOI:** 10.1038/s41467-019-14000-9

**Published:** 2020-01-08

**Authors:** Amy Y. Clark, Karen E. Martin, José R. García, Christopher T. Johnson, Hannah S. Theriault, Woojin M. Han, Dennis W. Zhou, Edward A. Botchwey, Andrés J. García

**Affiliations:** 1 Woodruff School of Mechanical Engineering, Georgia Institute of Technology, Atlanta, GA 30332 USA; 2 Petit Institute for Bioengineering and Biosciences, Georgia Institute of Technology, Atlanta, GA 30332 USA; 3 Coulter Department of Biomedical Engineering, Georgia Tech and Emory, Atlanta, GA 30332 USA

**Keywords:** Biomaterials, Regenerative medicine, Tissue engineering

## Abstract

Stem cell therapies are limited by poor cell survival and engraftment. A hurdle to the use of materials for cell delivery is the lack of understanding of material properties that govern transplanted stem cell functionality. Here, we show that synthetic hydrogels presenting integrin-specific peptides enhance the survival, persistence, and osteo-reparative functions of human bone marrow-derived mesenchymal stem cells (hMSCs) transplanted in murine bone defects. Integrin-specific hydrogels regulate hMSC adhesion, paracrine signaling, and osteoblastic differentiation in vitro. Hydrogels presenting GFOGER, a peptide targeting α2β1 integrin, prolong hMSC survival and engraftment in a segmental bone defect and result in improved bone repair compared to other peptides. Integrin-specific hydrogels have diverse pleiotropic effects on hMSC reparative activities, modulating in vitro cytokine secretion and in vivo gene expression for effectors associated with inflammation, vascularization, and bone formation. These results demonstrate that integrin-specific hydrogels improve tissue healing by directing hMSC survival, engraftment, and reparative activities.

## Abstract (web summary)

Mesenchymal stromal cells enhance bone and cartilage repair, but are limited by poor survival and retention after transplantation. Here, the authors show that synthetic hydrogels presenting integrin-specific peptides enhance the survival and persistence of human mesenchymal stromal cells after transplant, as well as bone repair.

## Introduction

Because of their capacity for self-renewal, potential for multipotent differentiation, hypoimmunogenicity, and ability to home to injured tissues^1,2^, mesenchymal stem cells (MSC), also referred to as multipotent mesenchymal stromal cells, are a promising cell source for diverse regenerative medicine applications with over 350 active clinical trials in 2015 in the United States of America^3,4^. In musculoskeletal applications, transplanted MSC enhance bone, cartilage, and intervertebral disc repair in pre-clinical models and clinical trials^1,3^. In addition, MSC secrete a myriad of cytokines, growth factors and metabolites that modulate innate and adaptive immune responses^2^. These immunomodulatory properties of MSC are being explored for therapeutic application in a variety of chronic inflammation and autoimmune diseases. Therapeutic success, however, has been severely limited by poor survival, retention (<2%) and engraftment as the majority of delivered MSC die or are washed away as quickly as 1 h post-transplantation^5–7^.

Biomaterial carriers have been extensively evaluated for improving MSC delivery and survival. Synthetic hydrogel systems are particularly promising due to their cytocompatibility, injectability, and versatility in presenting bioactive functionalities. Despite considerable efforts in engineering hydrogels to mimic characteristics of natural extracellular matrices (ECM), hydrogel carriers have not significantly impacted the clinical translation of MSC therapies. A key barrier to their translation is the lack of understanding of how hydrogel biophysical and biochemical properties influence the in vivo survival, retention, and function of MSCs. Work with modified alginate gels has identified elastic modulus and pore volume as biophysical cues that control MSC activities^8^. Although many hydrogels incorporate short peptides (e.g., RGD) to support integrin-mediated cell adhesion, the impact of integrin-specific signals on biomaterial delivered cell survival and function remains unclear.

Integrin αβ heterodimers, a major family of cell-ECM adhesion receptors^9^, transduce extracellular biophysical and biochemical signals regulating diverse cellular processes, including cell anchorage, migration, survival, lineage commitment, and expression of differentiated phenotypes^10–12^. MSCs express various integrin heterodimers that bind to fibronectin, type I collagen, laminins, and other ECM components^13^. Integrins play pivotal roles in the interactions of cells with biomedical devices and tissue engineering constructs, and controlling integrin binding has emerged as a promising strategy to direct MSC in vitro activities^14^. Previous work has shown that integrin binding specificity modulates MSC adhesion and differentiation in vitro^15–18^, but a fundamental understanding of the influence of integrin binding specificity on MSC functionality in vivo is lacking.

In this study, we investigate the impact of integrin-specific peptides on the survival, persistence, and reparative functions of human bone marrow-derived MSC (hMSC) delivered within a synthetic, degradable hydrogel to a non-healing bone defect. We demonstrate that hydrogels presenting integrin-specific peptides modulate hMSC activities in vitro and promote stem cell survival and engraftment within the bone defect to improve tissue repair. Integrin-specific hydrogels differentially potentiate the in vitro hMSC immunomodulatory secretome, as well as host reparative gene expression profiles in vivo. These results demonstrate that biomaterial integrin specificity can be used to direct hMSC survival, engraftment, and secretory and reparative activities during tissue healing.

## Results

### Integrin-specific hydrogels support in vitro hMSC activities

**Fig. 1:**
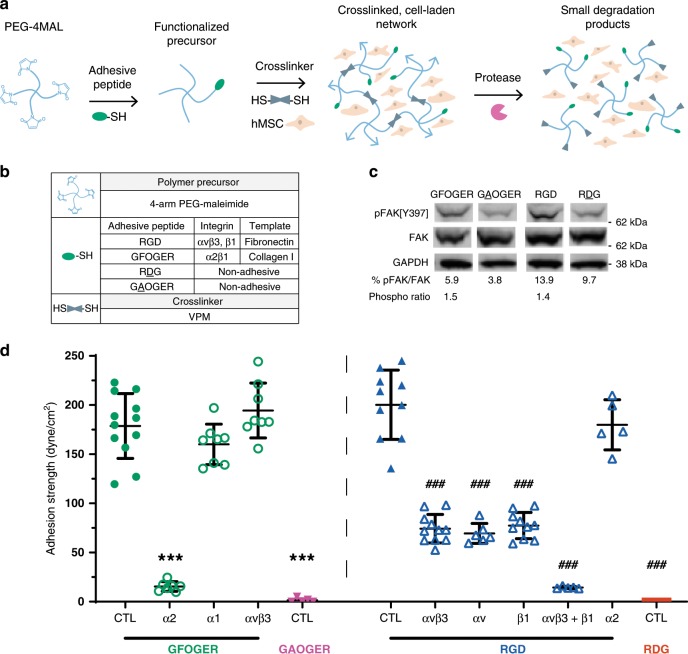
Integrin-specific peptide-functionalized hydrogels modulate cell adhesion. **a** 4-arm PEG-maleimide hydrogel reaction scheme (cells not shown to scale relative to hydrogel). **b** Hydrogel components. **c** Western blot for FAK phosphorylation indicates integrin activation of encapsulated hMSC. hMSCs exhibited 1.5- and 1.4-fold greater phosphorylation of FAK at Y397 when encapsulated in hydrogels functionalized with adhesive GFOGER and RGD peptides, compared with inactive peptides, GAOGER and RDG, respectively. For all blots, two-color imaging of the same membrane was performed using different species antibodies for FAK and pFAK, and the loading control GAPDH was run on the same membrane. Representative blots from three biologically independent runs are shown. **d** Adhesion strength values for hMSC adhering to hydrogels in the presence or absence of blocking antibody. Each point represents a biologically independent sample (sample size: GFOGER CTL = 12, GFOGER α2 = 7, GFOGER α1 = 8, GFOGER αvβ3 = 8, GAOGER CTL = 5, RGD CTL = 10, RGD αvβ3 = 11, RGD αv = 6, RGD β1 = 10, RGD αvβ3 + β1 = 5, RGD α2 = 5, RDG CTL = 4), mean ± SD. ANOVA (*p* < 0.0001) was used to detect statistical differences followed by Sidak’s multiple comparisons test with adjustment for multiple comparisons, ***p < 0.0001 vs. GFOGER CTL; ^###^*p* < 0.0001 vs. RGD CTL.

Poly(ethylene glycol) [PEG] hydrogels based on 4-arm PEG macromers with terminal maleimide groups (PEG-4MAL) were synthetized to present integrin-specific peptides. The PEG-4MAL platform allows for synthesis of structurally-defined hydrogels with independently tunable polymer density, adhesive peptide type and density, and crosslinker type and density^19,20^. Furthermore, PEG-4MAL exhibits excellent in vitro cytocompatibility and minimal inflammation and toxicity in vivo. Here, cysteine-terminated adhesive peptides were conjugated to the PEG-4MAL macromer via reaction with a maleimide group (Fig. 1a). These functionalized macromers were crosslinked into a network, with or without hMSC, by reacting them with a protease-degradable, cysteine-flanked crosslinker peptide. We examined two synthetic adhesive peptides with different integrin binding specificities (Fig. 1b). GFOGER is a triple helical synthetic peptide derived from type I collagen with high binding affinity for α2β1 integrin^21^. RGD is a short linear peptide present in fibronectin and other ECM proteins that binds several integrins, including αvβ3, αvβ1, and α5β1. hMSCs were isolated from human donor bone marrow and characterized by the NIH Resource Center at Texas A&M University and confirmed by surface marker expression as CD73^+^CD90^+^CD105^+^ and CD14^−^CD34^−^CD45^− ^(ref. ^22^) (Supplementary Fig. 1). They express α1, α2, α3, α5, α6, αv, β1, and β3 integrin subunits (Supplementary Fig. 2). As inactive control peptides, we used GAOGER (mutated GFOGER) and RDG (scrambled RGD) peptides. These inactive peptides are equivalent in size and conformation to their corresponding active peptides to allow for direct comparisons, as differences in peptide size/conformation could impact the nanoscale gel structure. Peptides were conjugated to PEG-4MAL with >98% tethering efficiency and there were no differences in tethered density among peptide type (Supplementary Fig. 3). For 4.5% polymer density gels, material properties such as storage modulus (~60 Pa) and mesh size (~30 nm) did not vary with respect to incorporated adhesive peptide type (Supplementary Fig. 4, Supplementary Table 1). In addition, for a fixed adhesive peptide density, the mechanical properties of the gel can be tuned by adjusting polymer density (Supplementary Fig. 5). These data demonstrate the ability of this synthetic hydrogel platform to present defined densities of different adhesive peptides while controlling for other material properties, a major advantage over other synthetic and natural hydrogel systems.

Integrins perform essential roles in the transduction of ECM properties through their association with focal adhesion proteins. An essential effector of this integrin-mediated signaling, focal adhesion kinase (FAK), regulates many cell processes including osteoblastic differentiation when FAK is phosphorylated at tyrosine-397 (ref. ^23^). We show that hMSCs cultured in GFOGER- and RGD-presenting hydrogels exhibited elevated FAK phosphorylation compared with their respective inactive peptide controls, while no difference in phosphorylation levels was seen between GFOGER- and RGD-presenting gels (Fig. 1c). The ability of these hydrogels to support hMSC adhesion was evaluated by measuring the adherent force of cells cultured on top of hydrogel disks. Using a hydrodynamic spinning disk assay, adherent cells were exposed to shear forces that increase linearly with radial position from the disk center, providing sensitive measurements of the force required to detach the cell from the substrate^24,25^. hMSCs were cultured on top of flat hydrogels with blocking or isotype control antibodies for 2 h, then the adhesion strength was measured (Fig. 1d). Both GFOGER- and RGD-presenting hydrogels supported high levels of adhesion strength, whereas the control GAOGER and RDG peptides supported background levels of adhesion. For GFOGER-presenting hydrogels, α2-blocking antibodies reduced the adhesion strength to background levels equivalent to GAOGER-presenting controls. In contrast, blocking either αVβ3 or α1 integrin had no effect on cell adhesion to GFOGER-presenting gels. This result demonstrates that α2 integrin mediates adhesion to GFOGER. The α2 subunit only dimerizes with β1 integrin, thus we conclude that hMSC adhesion to GFOGER-presenting gels is mediated by α2β1 integrin. For RGD-presenting gels, antibodies against αV, αVβ3, and β1 integrins individually reduced adhesion strength by ~60–65%, which is higher than background adhesion levels on RDG-presenting gels. Blocking both αVβ3 and β1 integrins completely eliminated adhesion to RGD-presenting gels. Importantly, α2-blocking antibody had no effects on the adhesion strength to RGD-presenting gels. These results show that αVβ3 and β1 integrins, but not α2β1 integrin, mediate cell adhesion to RGD-presenting gels. These results demonstrate that hMSC adhesion to GFOGER- (α2β1 integrin) and RGD- (αVβ3/β1 integrins) presenting gels is integrin specific. In addition, there is negligible hMSC adhesion to gels presenting the inactive peptides GAOGER and RDG.

**Fig. 2:**
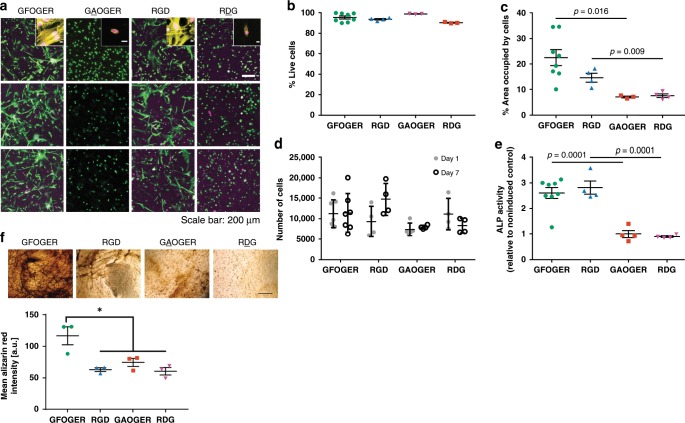
Integrin-specific peptide-functionalized hydrogels modulate in vitro behaviors of encapsulated hMSC. **a** Hydrogel-encapsulated hMSCs were cultured for 7 days and stained with Calcein-AM (green) and ethidium homodimer (magenta). Inset shows actin (yellow) and nuclei (magenta) stains. Scale bar 200 µm, 10 µm inset. **b** Quantification of viability staining indicates high viability (>90%) in all peptide-functionalized hydrogels. Each point represents a biologically independent sample (sample size: GFOGER = 8, RGD = 4, GAOGER = 3, RDG = 3), mean ± SE. **c** Quantification of spread cell area indicates significantly more spreading in GFOGER- and RGD-functionalized hydrogels compared with non-adhesive GAOGER- and RDG-functionalized hydrogels, respectively. Each point represents a biologically independent sample (Sample size: GFOGER = 8, RGD = 4, GAOGER = 3, RDG = 4), mean ± SE. Kruskal–Wallis test (*p* < 0.0001) was used to detect differences followed by Dunn’s multiple comparisons test, *p* < 0.016 GFOGER vs. GAOGER; *p* < 0.009 RGD vs. RDG. **d** Quantification of cell number by DNA content for hMSC-laden peptide-functionalized hydrogels cultured over 1 week. Each point represents a biologically independent sample (sample size: GFOGER = 7, RGD = 4, GAOGER = 5, RDG = 4), mean ± SD. Two-way ANOVA did not show differences (*p* = 0.44). **e** Alkaline phosphatase activity was quantified for hMSC encapsulated in peptide-functionalized hydrogels and cultured for 9 days in growth or osteogenic conditions. Each point represents a biologically independent replicate (sample size: GFOGER = 8, RGD = 4, GAOGER = 4, RDG = 4), mean ± SE. ANOVA (*p* < 0.0001) was used to detect statistical differences followed by Sidak’s multiple comparisons test with adjustment for multiple comparisons, *p* < 0.0001 GFOGER vs. GAOGER; *p* < 0.0001 RGD vs. RDG. **f** Alizarin red staining of mineral deposition by hMSC encapsulated in integrin-specific hydrogels after 14 days of culture in growth media. Representative images of Alizarin red stained hMSC-laden hydrogels (scale bar 100 µm) and quantification of the mean Alizarin red intensity in the images. *n* = 3 independent samples per group, mean ± SE; *p < 0.05. ANOVA (*p* < 0.0053) was used to detect statistical differences followed by Tukey’s multiple comparisons test.

We next assessed the in vitro cytocompatibility and osteogenic capacity of integrin-specific hydrogels. hMSCs were encapsulated within adhesive peptide-functionalized hydrogels and high viability (>90%) was maintained after 1 week in culture (Fig. 2a, b). Cytoskeletal actin labeling showed networks consisting of multiple, elongated cells with well-defined actin fibers within GFOGER- and RGD-functionalized hydrogels, whereas cells in hydrogels presenting non-adhesive peptides were round with diffuse actin staining (Fig. 2a, inset). hMSCs cultured in GFOGER- and RGD-presenting hydrogels exhibited higher spread area compared with hydrogels functionalized with the respective inactive controls GAOGER and RDG (Fig. 2c); no differences in spread area were observed between GFOGER- and RGD-functionalized gels. We next examined whether integrin-specific hydrogels modulate cell proliferation in 3D. No differences in initial cell loading (day 1) or cell number at 7 days in culture were noted among integrin-specific hydrogels (Fig. 2d). Analysis of cell proliferation via incorporation of 5-ethyl-2′-deoxyuridine showed equivalent proliferation rates among adhesive peptide-presenting gels with median values around 2–4% (Supplementary Fig. 6).

To examine osteogenic differentiation for hMSC encapsulated in integrin-specific hydrogels, alkaline phosphatase (ALP) activity, an early marker of osteogenic differentiation, was assessed for hMSC-laden hydrogels cultured in osteogenic or growth media. Cells within GFOGER- and RGD-functionalized hydrogels exhibited significant increases in ALP activity compared with cells cultured within GAOGER- and RDG-presenting hydrogels (Fig. 2e); no differences in alkaline phosphatase activity were observed between GFOGER- and RGD-functionalized gels. Mineral deposition, a late functional marker of osteoblastic differentiation, was evaluated in hMSC-laden hydrogels cultured for 14 days in growth media by staining with Alizarin red. GFOGER-presenting hydrogels supported higher levels of mineral deposition compared with RGD- and inactive adhesive peptide-presenting gels (Fig. 2f). The increase in mineral deposition for hMSC cultured within GFOGER-presenting hydrogels compared with RGD-functionalized gels is consistent with our results for cells cultured on top of these hydrogels^26^. These results demonstrate that GFOGER- and RGD-presenting hydrogels support robust 3D adhesion, signaling, and osteoblastic differentiation compared with GAOGER and RDG-functionalized gels.

### Integrin-specific gels prolong hMSC in vivo survival

**Fig. 3:**
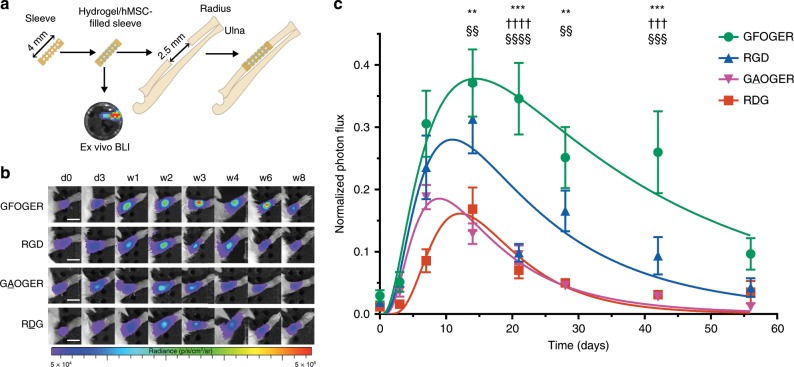
Adhesive peptide-functionalized hydrogels modulate transplanted hMSC survival and persistence. **a** Schematic of hydrogel implantation in radial segmental defect. A 4 mm perforated, polyimide sleeve was filled with hydrogel/hMSC before surgery and bioluminescence was measured. A 2.5 mm defect was created in the right radius of a mouse and the hydrogel-filled sleeve was fitted over the ends of the defect followed by in vivo bioluminescence imaging over the course of the study. **b** Representative bioluminescence images over time for each treatment group. Scale bar 5 mm. **c** In vivo bioluminescence signal for hMSC^FLuc^ delivered in hydrogels implanted into radial defects was monitored using IVIS, and photon flux at each time point was normalized to initial, pre-implantation flux of each implant. Sample size (mice): GFOGER = 13, RGD = 8, GAOGER = 6, RDG = 8, over two independent experiments; mean ± SE. Two-way repeated-measures ANOVA (peptide: *p* < 0.0001, time: *p* < 0.0001) was used to detect statistical differences followed by Sidak’s multiple comparisons test, **p < 0.001, ***p < 0.0001 GFOGER vs. GAOGER; ^§§^*p* < 0.01, ^§§§^*p* < 0.001, ^§§§§§^*p* < 0.0001 GFOGER vs. RDG; ^†††^*p* < 0.008, ^††††^*p* < 0.0001 GFOGER vs. RGD.

We next evaluated the ability of integrin-specific hydrogels to modulate the survival and persistence of transplanted hMSC in a non-healing segmental defect in the mouse radius. This bone repair model is ideally suited for screening therapeutic formulations and has significant advantages over other bone defect models, including reduced procedure times, no hardware fixation as the adjacent ulna stabilizes the defect, application of in vivo imaging techniques, and potential use of transgenic models. Importantly, this segmental defect is a critical sized defect that does not heal over the 8-week experimental time window but heals in response to therapeutic doses of BMP-2 (Supplementary Fig. 7). The radial 2.5-mm segmental defect is created using custom-built bone cutters. The hydrogel is cast within a polyimide sleeve laser-machined with holes to support nutrient transport and tissue ingrowth and is fitted within the defect (Fig. 3a). To monitor hMSC survival and persistence post-implantation, cells were transduced with a lentivirus to stably and constitutively express red firefly luciferase and tracked over time using in vivo bioluminescence imaging. Lentiviral transduction resulted in a 15% reduction in growth potential but did not alter osteogenic differentiation potential for transduced cells (hMSC^FLuc^) compared with unmodified hMSC (Supplementary Fig. 8).

Hydrogels loaded with early passage hMSC^FLuc^ were delivered to radial segmental defects in male NOD *scid* gamma (NSG) mice. The NSG mouse lacks mature B and T cells and is widely used for human cell transplantation studies^27^. Initial studies compared hydrogels of varying polymer densities (4.5%, 6.0%, 8.0% w/v) with fixed adhesive peptide density and gels of constant polymer density with varying adhesive peptide densities (0.3 mM, 1.0 mM) to identify hydrogel formulations that support bone formation. Hydrogel polymer density impacts bone formation as lower density gels supported increased bone volume compared with higher density gels (Supplementary Fig. 9). Adhesive peptide density modulates bone formation with higher bone volume for gels functionalized with 1.0 mM compared with 0.3 mM GFOGER (Supplementary Fig. 10). Based on these results, a 4.5% hydrogel with 1.0 mM adhesive peptide density was selected as the base hydrogel formulation for subsequent studies on hMSC-mediated repair.

hMSCs were encapsulated within hydrogels presenting equimolar densities of different adhesive peptides for delivery to the bone defect. Each implant contained 15,000 hMSC^FLuc^ with no differences in encapsulated cell density among adhesive peptide hydrogel formulations (Supplementary Fig. 11). Hydrogel constructs were scanned for bioluminescence immediately prior to implantation, immediately after implantation, and at selected time points post-implantation. Following transplantation, bioluminescence signal for all groups was significantly higher than the detection limit and localized to the transplant site (Fig. 3b). Clear differences in bioluminescence signal over time and among integrin-specific hydrogels were evident with higher normalized intensity and longer signal duration for hMSC^FLuc^ delivered in GFOGER-functionalized gels (Fig. 3b). To compare among groups, the peak bioluminescence signal, normalized to the pre-implantation (ex vivo) signal, was plotted as a function of implantation time (Fig. 3c). Bioluminescence signal rapidly increased at early time points to reach a maximum around day 14 post-transplantation and then decayed slowly down to background levels. Similar biphasic signal patterns have been reported for transplanted luciferase-expressing stem cells^28,29^. Bone defects treated with GFOGER- and RGD-functionalized hydrogels containing hMSC^FLuc^ exhibited significantly increased bioluminescence signal compared with control GAOGER- and RDG-presenting hydrogels with hMSC^FLuc^, which exhibited similar bioluminescence profiles (Fig. 3c). For each imaging time point, the time-to-peak bioluminescence signal post-luciferin injection was analyzed. The time-to-peak signal decreased from 50 min at early imaging days to reach stable values around 30 min after 7 days post-implantation (Supplementary Fig. 12). This early delay in time-to-peak bioluminescence is attributed to delayed transport of luciferin substrate to cells in the defect. This observation is not unexpected as a significant tissue volume is excised to create the segmental defect, which is then filled with an initially avascular gel within a polymeric sleeve. For quantitative comparisons among hydrogel formulations, we excluded earlier time points (<7 days) to avoid any confounding effects from luciferin transport limitations. Two-way repeated-measures ANOVA revealed differences among groups for adhesive peptide (*p* < 0.0001) and time (*p* < 0.0001). hMSC^FLuc^ in GFOGER-presenting hydrogels exhibited higher total bioluminescence signal compared with hMSC^FLuc^ in GAOGER- and RDG-functionalized gels for all time points except for day 56 post-transplantation (Fig. 3c). In contrast, there were no significant differences in bioluminescence signal between RGD- and RDG-presenting gels for any time point. Notably, hMSC^FLuc^ in GFOGER-functionalized gels exhibited higher bioluminescence signal compared with hMSC^FLuc^ in RGD-functionalized gels at days 21 and 42 post-transplantation. Immunostaining for human-specific nuclear mitotic antigen (NuMa) confirmed the presence of human cells in the bone defect at weeks 4 and 8, with the majority of staining localized in the bone marrow and not mineralized bone (Supplementary Fig. 13). Increased NuMA-positive staining was evident for defects treated with GFOGER-presenting hydrogels compared with GAOGER-, RGD- or RDG-functionalized hydrogels, consistent with bioluminescence data. These results demonstrate that GFOGER-functionalized hydrogels support enhanced hMSC survival and persistence within bone defects compared with RGD-presenting and non-adhesive hydrogels.

### Integrin-specific gels promote hMSC-dependent bone repair

**Fig. 4:**
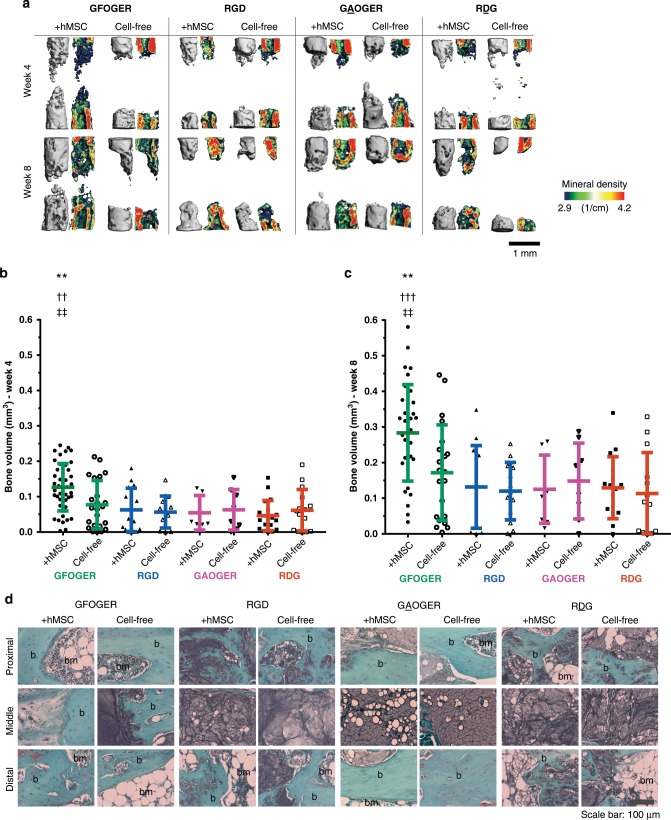
GFOGER-functionalized hydrogels enhance bone repair with hMSC transplantation. Peptide-functionalized hydrogels with or without encapsulated hMSCs were implanted into radial segmental defects in NSG mice. **a** Bone formation was monitored with live animal µCT. Representative 3-D reconstructions with sagittal mineral density heat maps. Scale bar 1.0 mm. Bone volume was quantified in the middle 2.0 mm of the original 2.5 mm defect at **b** week 4 and **c** week 8. Sample size (mice): GFOGER = 34 (+ hMSC), 22 (cell-free), RGD = 13 (+ hMSC), 13 (cell-free), GAOGER = 8 (+ hMSC), 8 (cell-free), RDG = 15 (+ hMSC), 13 (cell-free), over five independent experiments; mean ± SE. ANOVA (*p* < 0.0001) was used to detect statistical differences followed by Sidak’s multiple comparisons test, **p < 0.009 GFOGER vs. GAOGER; ^††^*p* < 0.001, ^†††^*p* < 0.0006 GFOGER vs. RGD; ^‡‡^*p* < 0.008, GFOGER + hMSC vs. GFOGER cell-free. **d** Safranin-O and fast green immunohistochemical staining of defects at weeks 4 and 8 (b: bone, bm: bone marrow). Scale bar 100 µm.

We explored whether integrin-specific hydrogels delivering hMSC modulate bone repair. Integrin-specific hydrogels with or without 15,000 hMSCs were delivered to the radial segmental defect of NSG mice as previously described. Newly formed bone was quantified by live animal micro-computed tomography (µCT) at 4 and 8 weeks (Fig. 4). No significant difference was observed in bone repair between unmodified hMSC and hMSC^FLuc^ (Supplementary Fig. 14), and these data sets were pooled. Figure 4a shows representative 3D reconstructions of a 3.2 mm segment encompassing the original 2.5 mm defect and sagittal cross-sections with a mineral density heat map overlay. Bone defects treated with RGD-, GAOGER- or RDG-functionalized hydrogels exhibited very low levels of new bone in the center of the defect, with no significant difference between hMSC-laden and cell-free hydrogels (Fig. 4a–c). In contrast, radial defects treated with hMSC in GFOGER-functionalized hydrogels exhibited significantly higher levels of new bone formation at both 4 and 8 weeks compared with defects treated with hMSC-laden RGD-presenting or inactive peptide-functionalized gels (ANOVA, *p* < 0.0001, Fig. 4b, c). Bone formation at both 4 and 8 weeks was higher in defects treated with hMSC-laden GFOGER-presenting gels compared with cell-free GFOGER-functionalized gels, demonstrating that the enhancements in bone formation resulted from the transplanted hMSCs. No differences in bone formation were observed among cell-free hydrogels.

Histological sections of treated bone defects at week 8 were stained with Safranin-O/fast green to visualize repair tissue (Fig. 4d). Bone marrow and lamellar bone were evident at the proximal and distal ends of all defects. The middle section of defects treated with hMSC-laden GFOGER-functionalized hydrogels shows high amounts of collagen-rich, bone-like tissue compared with all other groups, consistent with µCT results. The middle section of defects treated with GAOGER-, RGD-, and RDG-presenting hydrogels displays disorganized tissue with evidence of non-degraded hydrogel fragments. There was no staining for cartilage in any defects. Collectively, the µCT and histological results demonstrate increased hMSC-dependent bone repair for GFOGER-presenting hydrogels compared with other adhesive peptide groups. The differences in bone repair among integrin-specific hydrogels are in agreement with the observed differences in hMSC survival and persistence.

### Integrin-specific hydrogels modulate host gene expression

**Fig. 5:**
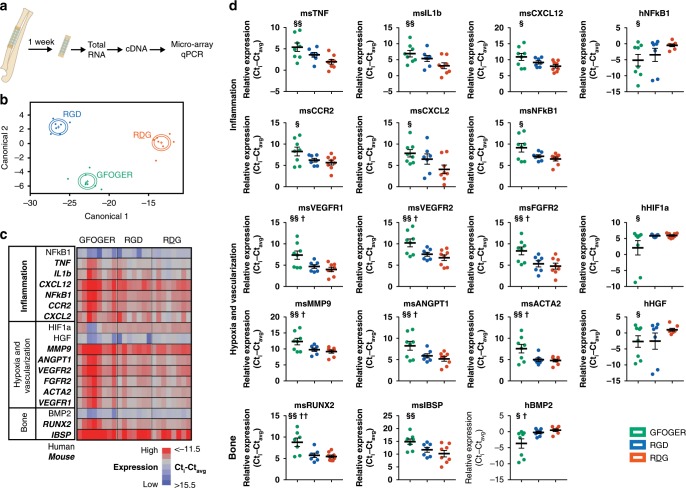
hMSCs in GFOGER-presenting gels result in upregulation of inflammation, vascularization and bone genes in vivo. **a** Following implantation of hMSC-laden hydrogels, tissue was explanted from the defect at 1 week and total RNA was extracted and standardized across all samples. **b** Linear discriminant analysis of gene expression profiles. Each point represents a mouse sample, “ + ” symbol corresponds to each multivariate mean, and ellipses represent a 95% confidence level—thus groups that differ significantly do not intersect. **c**, **d** Genes exhibiting significantly different expression levels with respect to ligand peptide. Mean normalized Ct values were used for the analysis and are represented in (**c**) the heat map where red is high expression and blue is low expression or (**d**) data plots (mean ± SE). Sample size (mice): GFOGER = 8, RGD = 7, RDG = 8. One-way ANOVA was used to detect statistical differences followed by False Discovery Rate analysis using two-state linear step-up procedure of Benjamini, Krieger, and Yekutieli, ^§^*p* < 0.05 GFOGER vs. RDG; ^§§^*p* < 0.01 GFOGER vs. RDG; ^†^*p* < 0.05, GFOGER vs. RGD; ^††^*p* < 0.01, GFOGER vs. RGD.

Based on the enhanced hMSC persistence and bone repair in radial segmental defects treated with hMSC delivered in GFOGER-functionalized gels, we hypothesized that integrin-specific hydrogels differentially modulate early inflammatory and reparative gene expression profiles within the defect. To examine this, hMSCs were delivered to radial defects in GFOGER-, RGD-, or RDG-functionalized hydrogels. Repair tissue within the defect was explanted 1 week after implantation and processed for RNA extraction (Fig. 5a). This early time point was selected based on previous reports showing significant differences in gene expression profiling at early time points (7–14 days) for bone repair models^30–32^. Ninety-six human and mouse gene targets related to vascularization, bone, inflammation, wound healing, matrix proteins, and cell survival were evaluated by microfluidic PCR analysis on the Fluidigm system. Of the 96 gene targets, ~60 genes resulted in a detectable signal above background (Supplementary Fig. 15). Linear discriminant analysis revealed clear separation of the samples when grouped by adhesive peptide type as shown in the canonical plot presenting the sample points and multivariate means on the two canonical axes that best separate the groups (Fig. 5b). The canonical plot shows the adhesive peptide groups GFOGER and RGD closer together and more removed from RDG along the canonical 1 axis, but with no overlap among the groups. Along canonical axis 2, RGD and RDG exhibit slight overlap whereas GFOGER is farther removed from the other two peptide groups, indicating more similarity between RGD and RDG than GFOGER and RGD or GFOGER and RDG. Due to space constraints in the microfluidic Fluidigm chip, samples for GAOGER-presenting hydrogels were not analyzed in the microfluidic platform. Nevertheless, direct comparisons using conventional qRT-PCR showed no significant differences in gene expression among the inactive control peptides RDG and GAOGER (Supplementary Fig. 16). This result showing no differences in gene expression between RDG- and GAOGER-functionalized hydrogels is full agreement with all other biological responses evaluated, including bone repair.

Supplementary Fig. 15 lists genes sorted by categorical function with red and blue indicating higher and lower normalized expression, respectively. This analysis identified significant differences among integrin-specific hydrogels for three gene clusters corresponding to inflammation, vascularization and hypoxia, and bone-related genes (Fig. 5c, d). Among host mouse genes, the inflammatory genes tumor necrosis factor (*TNF*), interleukin 1-beta (*IL1b*), stromal cell-derived factor 1 (*SDF1/CXCL12*), monocyte chemoattractant protein-1 receptor (*CCR2*), macrophage inflammatory protein 2-alpha (*MIP2A/CXCL2*), and nuclear factor-kappaB (*NFκB1*) were upregulated in GFOGER-presenting gels compared with the inactive peptide RDG-functionalized hydrogels. Human (from transplanted hMSC) *NFκB1* gene expression was upregulated in RDG-presenting gels compared with GFOGER-functionalized hydrogels. No differences in any inflammation-associated genes were detected between RGD- and RDG- or GFOGER- and RGD-functionalized gels. These differentially regulated inflammatory genes have been reported to influence the fracture-healing cascade through mediation of the early inflammatory response^33,34^, recruitment of lymphocytes and endothelial cells for vascular development^35^, leukocyte recruitment and activation^36,37^, and regulation of cellular stress^38^. Prolonged upregulation of these genes may lead to abnormal healing and persistent inflammation; however, transient increases in expression at 1 week after injury is in line with the normal fracture-healing cascade.

Several vascularization and hypoxia-related genes were differentially expressed in response to integrin-specific hydrogels. Six host mouse genes were upregulated in GFOGER-functionalized gels compared with RGD- and RDG-functionalized gels: VEGF receptor-1 (*VEGFR1/FLT1*), VEGF receptor-2 (*VEGFR2/KDR*), fibroblast growth factor receptor-2 (*FGFR2*), matrix metallopeptidase-9 (*MMP9*), angiopoietin-1 (*AGNPT1*), and α-actin-2 (*ACTA2*). Human hypoxia inducible factor-1α (*HIF1a*) and human hepatocyte growth factor (*HGF*) were downregulated in GFOGER-functionalized gels compared with RDG-presenting gels. The upregulated mouse vascularization genes have been implicated in angiogenesis and vascularization processes including cell migration^39^, sprouting^39,40^, tube formation^39^, vascular remodeling^39,40^, and vessel maturation and stabilization by pericytes^41^. The genes downregulated in transplanted hMSC in GFOGER gels are associated with cellular responses to hypoxic conditions and apoptosis^42,43^. In particular, HGF, a paracrine factor secreted by apoptotic cells, stimulates endothelial cell motility and growth and activates the NF-κB pathway which can then regulate HIF-1α expression^43,44^. Interestingly, human *HGF*, *NFκB1*, and *HIF1α* were all upregulated in hMSCs encapsulated in RDG-functionalized hydrogels compared with GFOGER-functionalized gels. The data show several angiogenic host genes upregulated in GFOGER-presenting gels, whereas hypoxic and apoptotic markers were upregulated in RGD- and RDG-functionalized groups. Although differences were observed in early vascularization gene expression, no gross differences were detected in functional vasculature levels within the defect at 8 weeks by µCT angiography between GFOGER- and RGD-functionalized hydrogels delivering hMSC to bone defects (Supplementary Fig. 17).

Expression of bone-related genes also showed differences among integrin-specific hydrogel groups. Murine Runt-related transcription factor-2 (*RUNX2*), a master transcription factor and regulator of osteoblastic genes^45^, was upregulated in GFOGER-presenting hydrogels compared with RGD- and RDG-functionalized gels. Murine bone sialoprotein (*IBSP*), which is regulated by Runx-2, was elevated in GFOGER-presenting gels compared with RDG-functionalized gels. In contrast, human bone morphogenetic protein-2 (*BMP2*) was downregulated in GFOGER-presenting hydrogels compared with RDG-functionalized gels. BMP-2 initiates the bone healing cascade and is critical for osteogenic differentiation and chondrocyte proliferation and maturation during endochondral bone development^46,47^. Hypoxia-triggered *BMP2* expression has been detected in human periosteum explants, supporting the observed simultaneous upregulation of human *HIF1A* and *BMP2* in hMSCs delivered by RDG-presenting hydrogels^48^. The upregulated expression of host bone formation-related genes in GFOGER-functionalized hydrogels is in agreement with the observed increased in vivo bone growth.

### Adhesive peptide shifts hMSC cytokine secretion profile

**Fig. 6:**
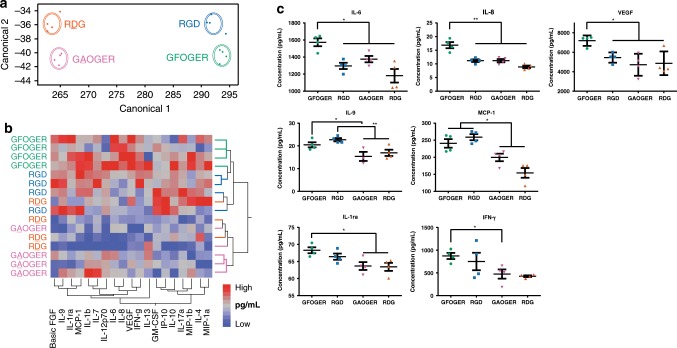
Integrin-specific peptide-functionalized hydrogels modulate in vitro cytokine secretome of encapsulated hMSC. Conditioned media from encapsulated hMSC in peptide-functionalized hydrogels was analyzed using bead-based multiplex technology for multiple cytokines. **a** Linear discriminant analysis of secreted cytokines from encapsulated hMSC. Each point represents a sample and each multivariate mean is a labeled circle corresponding to a 95% confidence limit for the mean. Groups that are significantly different have non-intersecting circles. **b** Hierarchical clustering using Ward’s method shows clustering of cytokine profiles from adhesive (GFOGER, RGD) hydrogels and non-adhesive (GAOGER, RDG) hydrogels. **c** Cytokines exhibiting significantly different secretion levels from hydrogel-encapsulated hMSC. *n* = 4 biologically independent samples, mean ± SE; *p < 0.05, **p < 0.01. One-way ANOVA was used to detect statistical differences followed by Tukey’s multiple comparisons test with adjustment for multiple comparisons.

A major mode of action of MSC is through paracrine effects of the MSC secretome. We therefore hypothesized that integrin-specific hydrogels differentially modulate the hMSC secretome resulting in the observed differences in early in vivo gene expression profiles and bone repair. We examined whether presentation of integrin-specific peptides within synthetic hydrogels alters the secretome of encapsulated hMSC in culture. hMSCs were encapsulated in GFOGER-, GAOGER-, RGD-, or RDG-functionalized hydrogels and cultured in growth medium for 48 h. Conditioned media was assayed for 27 human cytokines using Luminex multiplex technology (Supplementary Table 2). Linear discriminant analysis of the entire set of cytokines showed clear separation of the groups based on adhesive peptide type (Fig. 6a). Separation along canonical axis 1 resulted in cytokine profiles segregated based on adhesive peptide with GFOGER- and RGD-presenting hydrogels distinctly separated from non-adhesive GAOGER- and RDG-functionalized hydrogels. Separation along canonical axis 2 discriminated between GFOGER- and GAOGER-functionalized hydrogels and between RGD- and RDG-presenting hydrogels, although to a lesser degree than adhesive vs. non-adhesive groups. Consistent with this analysis, hierarchical clustering showed separation into one main cluster comprising GFOGER and RGD, and non-adhesive peptides GAOGER and RDG forming another cluster (Fig. 6b). Multivariate ANOVA (MANOVA) with a sum combination across cytokines revealed significant differences in cytokine secretion level among adhesive peptide groups (*p* < 0.001). A centroid canonical plot showed high similarity and overlap between GAOGER- and RDG-presenting hydrogels and a slightly shifted, but not significant, centroid profile for RGD-functionalized gels (Supplementary Fig. 18). Notably, the centroid for GFOGER-functionalized hydrogels is the farthest removed with minimal overlap, indicating that hMSC in GFOGER-presenting hydrogels secrete a significantly different cytokine profile compared with hMSC in RGD-, RDG-, or GAOGER-functionalized hydrogels, which showed no differences among each other.

Figure 6c presents results for individual cytokines that were differentially secreted among integrin-specific hydrogels. Interleukin-6 and -8 (IL-6, IL-8) and VEGF were elevated in GFOGER-functionalized hydrogels compared with RGD-, RDG-, and GAOGER-presenting hydrogels. IL-9 and monocyte chemotactic protein-1 (MCP-1) were elevated in GFOGER- and RGD-presenting hydrogels compared with their respective inactive peptide controls GAOGER- and RDG-functionalized gels. Interleukin-1 receptor antagonist (IL-1ra) and interferon-γ IFNγ) were upregulated in GFOGER-presenting hydrogels compared with GAOGER-functionalized hydrogel controls. These cytokines play diverse immunomodulatory roles including recruitment and activation of neutrophils (IL-8)^49^, recruitment of monocytes (MCP-1)^36,37,49^, polarization of monocytes toward alternatively activated, anti-inflammatory macrophages (IL-6)^50^, antigen-independent T-cell regulation (IL-9)^51^, and activation of multiple inflammatory cascades (IFNγ)^49,52^. In addition to immunomodulatory factors, the secretion of VEGF, a potent vasculogenic factor^53^, was also modulated. Collectively, these results demonstrate that the GFOGER peptide shifts the secretome of hydrogel-encapsulated hMSC relative to the other hydrogel-incorporated peptides.

To assess the functional impact of this difference in hMSC secretome among integrin-specific hydrogels, we performed a co-culture experiment to examine interactions between hydrogel-encapsulated hMSCs and M1-polarized macrophages (Supplementary Fig. 19). Primary murine monocytes were isolated from C57BL/6J mice, differentiated into macrophages for 7 days using M-CSF, and polarized towards an M1 inflammatory phenotype for 24 h using IFNγ and LPS. We chose to use murine macrophages due to their closer relation to our in vivo model and because they allowed us to assay for macrophage-specific cytokine secretion. As shown for hMSC-only controls (Supplementary Fig. 19a), there is minimal cross-reactivity between the hMSC-secreted human cytokines and the mouse multiplex cytokine array. hMSC were encapsulated in integrin-specific hydrogels and cultured for 48 h before co-culture with the macrophages. After 72 h of co-culture, conditioned media was assayed for 23 mouse cytokines using Luminex multiplex technology, and 12 cytokine targets showed higher-than-background readings. Hierarchical clustering using Ward’s method showed clustering of the cytokines based on adhesive peptide (Supplementary Fig. 19a). Linear discriminant analysis revealed clear separation of the groups based on adhesive peptide (Supplementary Fig. 19b). Co-culture of hMSCs encapsulated in GFOGER, RGD, and GAOGER gels with macrophages significantly downregulated macrophage secretion of the inflammatory cytokines RANTES (CCL5) and IL-1α compared with RDG gels. Co-culture of hMSC-laden GFOGER-functionalized gels and macrophages upregulated macrophage secretion of the cytokine IL-10, a potent anti-inflammatory factor, compared with all other gel conditions. hMSCs have been shown to promote IL-6 dependent macrophage polarization toward an anti-inflammatory IL-10 producing phenotype^50,54^, consistent with the increased hMSC secretion of IL-6 and increased macrophage secretion of IL-10 seen in our experiments. These results indicate that integrin-specific hydrogels alter the hMSC secretome and result in functional differences in hMSC-immune cell interactions.

## Discussion

We demonstrate that biomaterial integrin-specificity modulates the survival, engraftment, and reparative functions of bone marrow-derived hMSC transplanted in a non-union bone defect. To our knowledge, this is the first report demonstrating that biomaterial integrin-specificity modulates hMSC secretory and reparative activities in relevant in vivo models. Blocking studies show differences in integrin specificity for hMSC binding to GFOGER- (α2β1 integrin) and RGD- (αVβ3/β1 integrins) presenting gels, whereas the inactive peptide controls GAOGER and RDG support minimal cell adhesion. In order to evaluate the effects of biomaterial integrin specificity on hMSC activities, equimolar densities of adhesive peptides were used to engineer hydrogels with equivalent structural, mechanical, and biochemical properties but different integrin binding specificities. The number of bound integrin receptors depends on receptor type and expression, binding affinity, and ligand density, making it technically challenging to control for the number of bound receptors. Nevertheless, the equimolar peptide density used elicited equivalent short-term cell adhesive responses between GFOGER and RGD.

We show that presentation of the GFOGER peptide, but not RGD, significantly enhances transplanted hMSC survival, engraftment, and bone repair. We tuned PEG-4MAL hydrogel mechanical properties (*G*′ = 60 Pa) and adhesive peptide density (1.0 mM) to maximize radial defects repair. This gel formulation is considerably softer than RGD-presenting alginate gels (60 kPa) that supported bone formation in a calvarial defect^8^. This discrepancy in optimal elastic modulus likely reflects differences in gel structure and degradability that impact cell traction and morphological changes. Burdick et al. demonstrated that hMSC osteogenic differentiation is directed by the generation of degradation-mediated cellular traction, independently of cell morphology or matrix mechanics^55^. In addition, the hydrogel must allow for sufficient tissue ingrowth for successful healing either by its degradability or microporosity^8,56,57^.

We demonstrate that biomaterial integrin-specificity modulates the hMSC secretome and hMSC-macrophage interactions in vitro as well as host inflammation, vasculogenic, and osteogenic gene expression patterns in vivo, suggesting that hMSC paracrine secretions drive the observed differences in bone healing. The localization of hMSC to the bone marrow, not new bone tissue, further supports this model. The complimentary expression patterns of vasculogenic and monocyte related host genes in vivo and hMSC cytokines in vitro in GFOGER-presenting hydrogels is especially notable. In GFOGER-presenting hydrogels, hMSC-secreted elevated levels of VEGF in vitro. Host gene transcripts for the corresponding VEGF receptors, FGFR2, VEGFR1, and VEGFR2, were significantly upregulated in vivo. hMSC secretion of monocyte recuritment cytokine MCP-1 and IL-6 were also increased in vitro. In agreement with previous studies, IL-6 secreting hMSC promoted macrophage polarization toward an anti-inflammatory IL-10 producing phenotype in vitro^50,54^. The host receptor for MCP-1, CCR2, was also upregulated in GFOGER-presenting hydrogels in vivo. The significant interest in applying the MSC secretome therapeutically, including current clinical trials for inflammatory bowel disease and graft vs. host disease among others, make these findings particularly significant to the field.

We anticipate that our findings that hydrogel integrin specificity controls transplanted hMSC survival, engraftment, and reparative activities will impact fundamental studies on stem cell-material interactions as well as the engineering of delivery vehicles for cell-based therapies. The immunocompromised NSG mouse model used in these studies was necessary to assess hMSC survival and activities while avoiding xenograft rejection. A limitation of this model is the lack of a functional immune system, which influences bone healing responses. We considered evaluating murine MSC transplanted into immunocompetent mice; however, fundamental differences in both immune and MSC functions exist between mice and humans, limiting the translatability of mouse studies to human patients^58,59^. The evaluation of biomaterial integrin-specificity in hMSC-dependent bone repair in immunocompetent models (e.g., humanized mice) is a critical next step for the translation of this biomaterial strategy towards clinical applications.

## Methods

### Hydrogel synthesis

Peptide sequences and hydrogel components are listed in Table 1. 20 kDa PEG-4MAL (Laysan Bio), adhesive and control peptides (GFOGER, RGD, RDG, GAOGER), VPM crosslinker peptide, and dithiothreitol (DTT) in 100 mM HEPES in PBS, pH 6.5 were used. PEG-4MAL hydrogels were synthesized by reacting PEG-4MAL macromer with adhesive peptide, a 75:25 crosslinker mixture of VPM:DTT, and cell suspension (or buffer) at a volume ratio of 2:1:1:1 at the required concentrations to obtain the desired final concentration of PEG-4MAL and adhesive peptide. The concentration of crosslinker used for the synthesis of each hydrogel was calculated to stoichiometrically balance the number of free thiols on the crosslinker with the number of free (unreacted) maleimide groups remaining in the adhesive peptide-functionalized PEG-4MAL solution. Unless specified differently, hydrogels (25 µL) were cast as disks (4.5 mm diameter, 1.6 mm thickness) using silicone isolators (Grace Bio-Labs).

**Table 1:** Peptide hydrogel components.

Abbreviated name	Peptide sequence	Source
VPM	GCRDVPMSMRGGDRCG	AAPPTEC, Genscript
RGD	G**RGD**SPC	AAPPTEC
RDG	G**RDG**SPC	AAPPTEC
GFOGER	GGYGGGP(GPP)_5_**GFOGER**(GPP)_5_GPC, O = hydroxyproline	AAPPTEC, Genscript, Activotec UK
GAOGER	GGYGGGP(GPP)_5_**GAOGER**(GPP)_5_GPC, O = hydroxyproline	AAPPTEC

### hMSC

hMSCs were acquired from the NIH Resource Center at Texas A&M University. Cells were obtained under Texas A&M University IRB-approved protocols with informed consent from all human participants following relevant ethical regulations, and provided as de-identified frozen samples. Briefly, cells were obtained from healthy donors via bone marrow aspirate, followed by density centrifugation for mononuclear cells and selected for adherent culture. Cells were screened for colony forming units, cell growth, and differentiation into fat and bone using standard assays. Received frozen stocks were thawed and grown in MEM-α containing 16% fetal bovine serum (FBS), 2 mM L-glutamine and 100 U/mL penicillin/streptomycin (ThermoFisher, MA). Flow cytometry analyses confirmed that cells were positive for CD73, CD90, CD105, and negative for CD34, CD11b, CD45, CD19. hMSC were sub-cultured at 70–80% confluency, and for all experiments early passage (< 6) cells were used. hMSC from three donors were used in this study, and no significant differences were observed among donors.

### Adhesion studies

Glass coverslips (No. 1, 25 mm diameter) were plasma cleaned for 30 s then immersed in 5% v/v (3-mercaptopropyl)trimethoxysilane in toluene for 4 h at room temperature. Thiolated coverslips were then rinsed with toluene and 70% isopropanol, dried using a nitrogen gun, and stored under N_2_ (no more than 48 h) prior to use. Hydrogel components were mixed as described above (final volume 150 µL) and pipetted into a silicon isolator (20 mm diameter, 0.5 mm depth) sealed on top of Sigmacote-coated 25 mm diameter cover glass. A thiolated coverslip was then quickly inverted, thiol-side down, on top of the hydrogel solution. Following polymerization, the hydrogel disk bound to the thiolated coverslip was removed from the silicon isolator and swollen in PBS overnight at 4 °C.

hMSCs were incubated with integrin blocking, isotype control, or without antibody for 30 min prior to seeding on top of the hydrogel disks (1 × 10^4^ hMSCs/cm^2^). Pilot experiments were performed with different antibody concentrations to select concentrations with saturating effects for the experiments. Supplementary Table 3 lists antibodies and dilutions used. After 2 h, hMSC adhesion strength to the hydrogel disk was measured using a spinning disk device as described previously^25^. Hydrogel substrates with adherent cells were spun in PBS + 2 mM dextrose for 5 min at a constant speed. The applied shear stress (*τ*) is given by the formula

1 $$\tau = 0.8r\sqrt {\rho \mu \omega ^3}$$

where *r* is the radial position from the center of the sample and *ρ*, *µ*, and *ω* are the fluid density, viscosity, and rotational speed, respectively. After spinning, cells were fixed in 3.7% formaldehyde, permeabilized in 1% Triton X-100, stained with ethidium homodimer-1 (ThermoFisher) and counted at specific radial positions using a ×10 objective lens in a Nikon E400 microscope equipped with a Ludl motorized stage, Spot-RT camera and Image-Pro analysis software. A total of 61 fields were analyzed at prescribed locations and cell counts were normalized to the number of cells in the center of the disk. The fraction of adherent cells (*f*) was then fitted to a sigmoid curve

2 $$f = \frac{1}{{(1 + e^{\left[ {b\left( {\tau - \tau _{50}} \right)} \right]})}}$$

where *τ*_50_ is the shear stress for 50% detachment and *b* is the inflection slope. *τ*_50_ represents the mean adhesion strength for a population of cells.

### FAK phosphorylation

hMSCs were encapsulated and cultured in adhesive peptide-functionalized hydrogels overnight. Cells were washed twice with PBS and lysed by sonication on ice in cell extraction buffer containing protease and phosphatase inhibitors (ThermoFisher). The lysates were cleared by centrifugation at 10,000 rpm for 15 min at 4 °C and the extract was stored at −80 °C until analysis. Protein concentration was determined using a micro BCA kit (Pierce). Equivalent amounts of reduced, boiled (10 min at 70 °C) lysate were loaded on Bolt 10% Bis-Tris Plus gels (ThermoFisher) and subsequently transferred onto PVDF membranes. Membranes were probed with mouse monoclonal antibody against GAPDH (Abcam), mouse monoclonal antibody against FAK and rabbit polyclonal antibody against FAK [pY397] (ThermoFisher) at a 1:1000 dilution in 5% BSA TBS-T solution followed by fluorescent secondary antibodies (Li-Cor). Immunoblots were visualized on a Li-Cor Odyssey imaging system and analyzed using Image Studio Lite (Li-Cor) (Supplementary Fig. 20).

### Viability studies

hMSC-laden hydrogels were cultured free-floating in media and at specified time points, stained with Calcein-AM (ThermoFisher) for live hMSCs and ethidium homodimer (Life Technologies) for dead hMSCs. Gel-encapsulated hMSCs were visualized with a Nikon C2 laser scanning confocal head on a Nikon Eclipse-Ti microscope and Elements software (Nikon). Maximum projections on z-stacks were analyzed using ImageJ (NIH).

### Cell number

At specified time points, hydrogels were incubated in 1.0 mg/mL collagenase, type I (ThermoFisher) at 37 °C until fully degraded. Cells were lysed by sonication and freeze-thaw cycles. Whole cell lysate was assayed for DNA content and cell number using a CyQuant kit according to manufacturer’s instructions (ThermoFisher) and a cell standard curve.

### Cell spreading

A confocal microscope head (Nikon C2) and inverted microscope (Nikon Eclipse Ti) were used to acquire z-stacks (5–10) for each gel. Maximum intensity projections were created, and the percent of available area occupied by cells (% spread area) was calculated for each maximum intensity projection. % spread area for each biological replicate was the average of the areas for all of the maximum intensity projections for that gel.

### Osteogenic differentiation

hMSCs were seeded at 5 × 10^6^ cells/mL within integrin-specific hydrogels and cultured in osteogenic medium (Lonza). After 9 days of culture in induction medium, hMSCs were lysed and assayed for alkaline phosphatase activity (ALP) by incubating with 4-methylumbelliferyl phosphate disodium salt (MUP) substrate^60^. Hydrogels were incubated in 1 mg/mL collagenase type I (ThermoFisher) at 37 °C until fully degraded. Cells were resuspended in 50 mM Tris HCl (pH 7.4) and lysed by sonication and freeze-thaw cycles. Samples and ALP standards were loaded into a 96-well plate, then incubated with 60 μg/mL MUP substrate at 37 °C for 1 h and read at 360 nm excitation/465 nm emission. Enzymatic activity was standardized using purified calf intestinal ALP at known dilutions and normalized to total protein. For mineralization studies, hMSC-laden hydrogels were fed every 3–4 days with hMSC growth media. On day 14, gels were fixed with 10% neutral buffered formalin, stained with 2% Alizarin red solution for 30 min, and washed repeatedly with DI water until dye stopped leaching out of the gel. Gels were imaged using an Evos XL Core microscope (ThermoFisher). Mean Alizarin red intensity was calculated using ImageJ software.

### Luciferase lentiviral production

Lentiviral production was performed by the Viral Vector Core in the Neuroscience NINDS Core Facilities at Emory University. HEK 293FT (Invitrogen) cells were maintained in complete medium (DMEM, 10% FBS and 1% pen-strep), incubated at 37 °C, 5% CO_2_ and seeded at 70–80% confluence 1 day prior to transfection. HEK cells were incubated with transfection mixture (500 μg pLenti-UbC-RFLuc-tdtomato (Targeting Systems), 250 μg pMDLg/pRRE, 125 μg pRSV-REV187 and 150 μg pVSVG in ddH_2_O with 125 mM CaCl_2_ and 30 mM HEPES) for 7 h before fresh medium change. Lentivirus was harvested 72 h post-transfection by centrifuging the supernatant at 500 × *g* for 5 min at 40 °C, followed by passage through a 0.45 μm low protein binding filter. Filtered supernatant was then centrifuged at 91,000 × *g* for 2 h at 40 °C in a 45Ti rotor (Beckman). The virus pellets were re-suspended in 500 μL PBS, and after addition of 20% sucrose as a cushion, centrifuged at 91,000 × *g* for 2 h at 40 °C in a SW 41 rotor (Beckman). The virus pellet was resuspended in 100 μL PBS and stored at −80 °C.

### Lentiviral transduction

Transduction protocol was adapted from Lin et al.^61^. Early passage hMSCs (< 3) were seeded at 60–70% confluence and allowed to attach overnight. Media was replaced with a small volume of complete media containing 100 µg/mL protamine sulfate or 8 µg/mL Polybrene. Lentivirus was thawed on ice and added to the cells at MOI 5-20. Eight hours after initial infection, additional complete media with protamine sulfate was added to the plate, and 24 h after initial infection, media was replaced with fresh complete media. Six days after initial infection, transduction efficiency was measured by tdTomato expression by flow cytometry (BD Accuri C6). A scatter plot comparing 533 and 585 nm fluorescence emission was used in order to gate out the high auto-fluorescent cell population found within hMSCs.

### Implant preparation

Implant hydrogels (3 μL) were cast within 4-mm long polyimide tube sleeves (Microlumen) with laser machined 300-μm diameter holes to improve nutrient transport and cell invasion into the defect. All hydrogels used for in vivo studies contained 4.5% PEG-4MAL and 1.0 mM adhesive peptide (unless otherwise stated). All implant and hydrogel components were tested for endotoxin contamination and were confirmed to be below 0.1 EU/mL (5-fold lower than the US Food and Drug Administration’s recommended 0.5 EU/mL) by Limulus Amebocyte Lysate colorimetric assay (Lonza).

### Radial segmental defect surgery

All animal experiments were performed with the approval of the Georgia Tech Animal Care and Use Committee with veterinary supervision and within the guidelines of the Guide for the Care and Use of Laboratory Animals. NOD.Cg-*Prkdc*^scid^ *Il2rg*^tm1Wjl^/SzJ (NSG) male mice (8–10 weeks old, Jackson Laboratories) were anesthetized under isoflurane, and fur was removed from both forelimbs. The right forelimb was then swabbed with chlorohexidine and alcohol, and a 1.5-cm incision was made in the skin. Muscle tissue overlying the ulna and radius were blunt dissected, and 2.5 mm defects were made in the right radius using a custom-built bone cutter, while leaving the ulna intact. Implants were placed into the defect by fitting the polyimide sleeve over the radius at the proximal and distal ends of the defect holding the hydrogel in contact with the defect ends. The incision was then closed with Vicryl suture. Mice were given a single dose of slow-release buprenorphine for pain relief and were monitored post-surgery for signs of distress, normal eating habits and movement.

### Cell tracking in vivo

Bioluminescence of transplanted hMSC^FLuc^ was measured using an IVIS Spectrum CT (Perkin Elmer). Luciferin salt (Promega) was dissolved in physiological saline and sterile filtered through 0.22 μm pore membranes. Mice received a 150 mg/kg luciferin dose injected into the intraperitoneal cavity. Time to peak signal intensity was determined for each time point and 2D bioluminescence images were acquired 20–60 min post injection and analyzed with Living Image software (Perkin Elmer). Background bioluminescence of the unoperated arm was subtracted from the signal in the defect and signal is reported as photon flux, which normalizes for acquisition settings and ROI area.

### Faxitron and live animal µCT

X-ray images and 3D µCT images were acquired as previously described^26^. Briefly, radial defects were imaged with the MX-20 Radiography System (Faxitron). For µCT scanning, a 3.2 mm length of the radius centered about the 2.5 mm radial defects was scanned in anesthetized, live subjects using a VivaCT system (Scanco Medical, 145 mA intensity, 55 kVp energy, 200 ms integration time, and 15 µm resolution). Bone formation was evaluated by contouring 2D slices to include only the radius and applying a Gaussian filter. 3D µCT reconstructions display the full 3.2 mm length of radius scanned. However, in order to ensure that only new bone formation was measured, quantification of bone volume and mineral density within the defect was performed by evaluating only the middle 2.0 mm of the original 2.5 mm defect.

### Histology and immunostaining

Animals were euthanized 8 weeks after surgery by CO_2_ inhalation and their radii and ulna were harvested. Soft tissue was removed carefully without disturbing the defect and the bones fixed in 10% neutral buffered formalin overnight. Samples were briefly rinsed in tap water and decalcified in formic acid for 2 days. The samples were processed for paraffin embedding and sectioned to 5-μm thickness. For histological staining, sections were deparaffinized and hydrated. Sections were then stained with Weigert’s Iron Hematoxylin, 0.02% Fast Green, and 1.0% Safranin-O.

### RNA isolation and cDNA purification

Radial segmental defects in 8–10-week-old male NSG mice (Jackson Lab) were treated with 4.5% hydrogels functionalized with 1.0 mM adhesive peptide and crosslinked with 75:25 VPM:DTT with 15k hMSC (*n* = 7–8). The tissue within the 2.5 mm defect space was explanted at 1 week post-transplantation and stored in RNAlater solution (Qiagen) until further processing. Samples were placed in Qiazol solution (Qiagen), lysed by probe sonication, and homogenized in QIAshredder columns (Qiagen). Total RNA was isolated using an RNAeasy Plus Micro kit (Qiagen), and RNA content and purity were measured by spectrophotometry (NanoDrop 1000). cDNA synthesis was performed on total RNA (100 ng) using the High-Capacity RNA-to-cDNA Kit (ThermoFisher).

### qPCR microarray

Quantitative PCR was performed using Fluidigm 96 × 96 nanofluidic arrays targeting a set of 96 transcripts (human or murine) to observe changes in bone, survival, inflammation, vascularization, and matrix markers. Primers used are listed in Supplementary Table 4. The genes were pre-amplified in a single 13-cycle PCR reaction for each sample with EvaGreen Mastermix (Fluidigm BioMark) following the manufacturer’s protocol. Sixty-three gene targets resulted in detectable qPCR results. All subsequent statistical analyses were carried out using JMP-Genomics (SAS Institute) using the basic gene expression workflow^62^. Raw Ct values were imported into JMP-Genomics and normalized to mean Ct values across all genes for each sample for principal components analysis (PCA), assessment of the biological principal variance component contributions (PVCA), and hierarchical clustering using Ward’s method to identify sub-types of expression profile. Finally, one-way ANOVA was used to detect statistical differences followed by False Discovery Rate analysis using two-state linear step-up procedure of Benjamini, Krieger and Yekutieli. Results are presented as raw Ct values normalized to mean Ct values across all genes for a sample.

### Cytokine analysis in vitro

Early passage (< 6) hMSCs were encapsulated in 25 μL 4.5% hydrogels functionalized with 1.0 mM GFOGER, RGD, RDG, or GAOGER and crosslinked with 75:25 VPM:DTT. Following overnight culture, hydrogels were transferred to new wells and media was conditioned for 48 h. Conditioned medium was collected, supplemented with Halt protease inhibitor (ThermoFisher), and centrifuged at 10,000 × *g* for 10 min at 4 °C to remove debris. Supernatant was frozen in liquid nitrogen and stored at −80 °C until analysis. Conditioned media were analyzed using the Bio-Plex Pro Human Cytokine 27-plex Assay (Bio-Rad) on a Magpix multiplexing machine (Luminex) according to the manufacturer’s instructions. Multiple comparisons for secretion levels for each cytokine were performed using Fisher’s LSD test as only three comparisons were considered: GFOGER vs. RGD, GFOGER vs. GAOGER, and RGD vs RDG. Multivariate analyses were performed in JMP Pro v11.

### Statistical analysis

All experiments were performed on biological replicates. Sample size for each experimental group and statistical test used to determine significant differences among groups are reported in the appropriate figure legend. Non-parametric tests were used if the data did not meet the assumption of tests (e.g., non-normal data, different variances). For in vitro experiments, sample size was not pre-determined, and all samples were included in the analysis. For animal experiments, minimum sample size was determined based on power calculations to detect 20% differences among means using variances from previous/pilot experiments. All animals were used for analysis unless the mice died or had to be euthanized when found to meet pre-defined euthanization criteria (significant weight loss, unresponsive to external stimuli) according to the IACUC-approved animal protocol. The investigators were not blinded to outcome assessment and no randomization was used.

### Reporting summary

Further information on research design is available in the Nature Research Reporting Summary linked to this article.

## Supplementary information

Supplementary Information

Reporting Summary

## Data Availability

All materials are available either commercially or upon request. PCR array data is available from the Gene Expression Omnibus (GEO) database with accession code GSE141517.
