# Supplementary material for: Integrin-specific hydrogels modulate transplanted human bone marrow-derived mesenchymal stem cell survival, engraftment, and reparative activities
Source: Nat Commun. 2020 Jan 8;11:114. doi: 10.1038/s41467-019-14000-9 (PMC6949269; doi:10.1038/s41467-019-14000-9)
Supplement: Supplementary file 1 — Supplementary Information [file 41467_2019_14000_MOESM1_ESM.pdf]

SUPPLEMENTARY INFORMATION

Integrin-specific hydrogels modulate transplanted human mesenchymal stem cell survival, engraftment, and reparative activities

Clark et al.

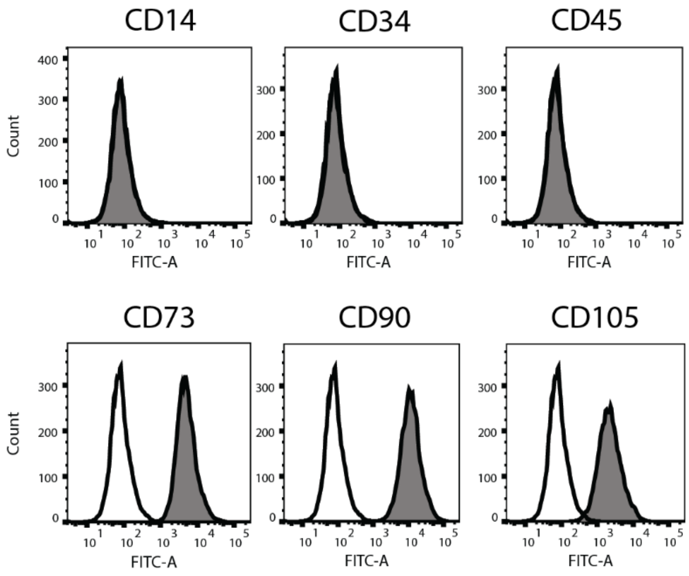

Supplementary Fig. 1. Expression profiles for human MSC markers evaluated by flow cytometry. Histograms for cells evaluated by surface marker-specific (dark fill) and isotype control (open) antibodies. Cells are positive for CD73, CD90, and CD105, and negative for CD14, CD34, and CD45.

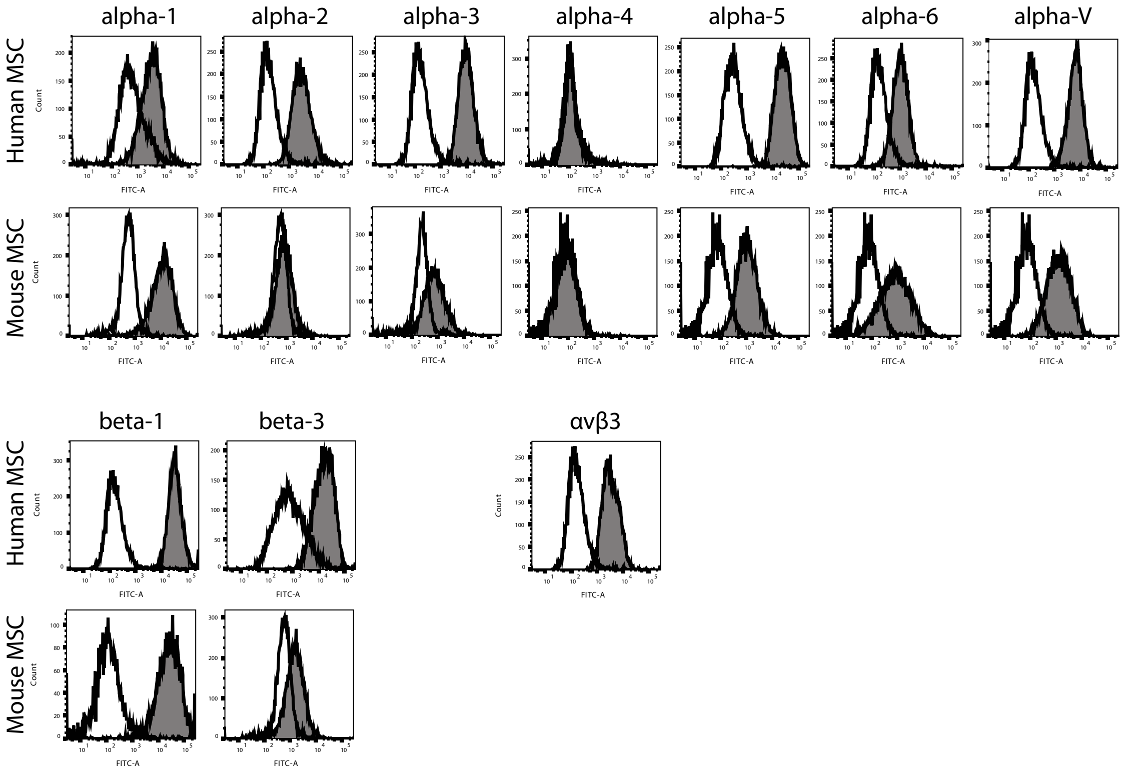

Supplementary Fig. 2. Integrin expression profiles for human and murine MSC evaluated by flow cytometry. Histograms for cells evaluated by integrin unit-specific (dark fill) and isotype control (open) antibodies.

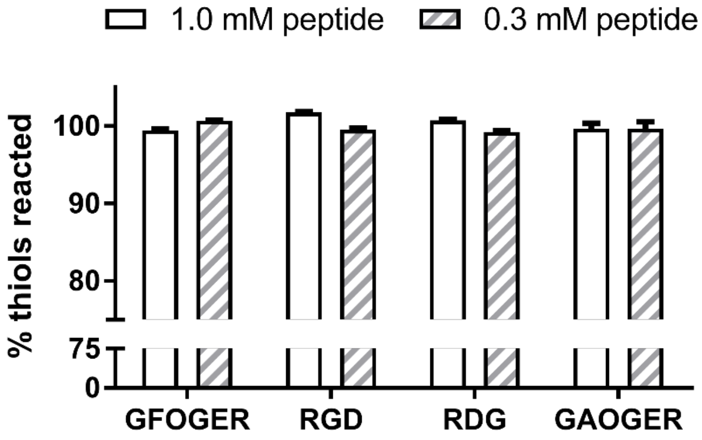

Supplementary Fig. 3. Adhesive peptides exhibit equivalent reactivity with PEG-4MAL macromer. Quantification of free thiols in solution following reaction of PEG-4MAL and peptide at two different ligand concentrations indicates virtually all peptide reacted with PEG-4MAL. n = 4 biologically independent samples; mean ± SE. One-way ANOVA was used to detect statistical differences ($p = 0.063$).

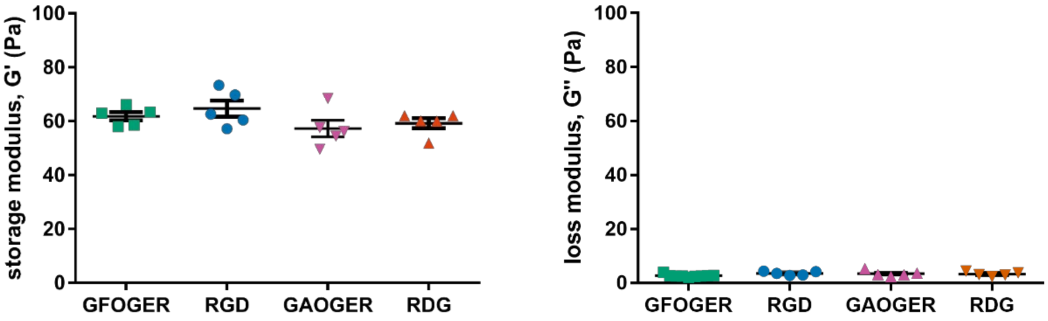

Supplementary Fig. 4. Adhesive peptide-functionalized hydrogels exhibit equivalent mechanical properties. Storage and moduli of hydrogels determined by rheological measurements. Each point represents a biologically independent sample (Sample size: GFOGER = 6, RGD = 5, GAOGER = 5, RDG = 5), mean ± SE. ANOVA showed no differences among groups ($p = 0.211$).

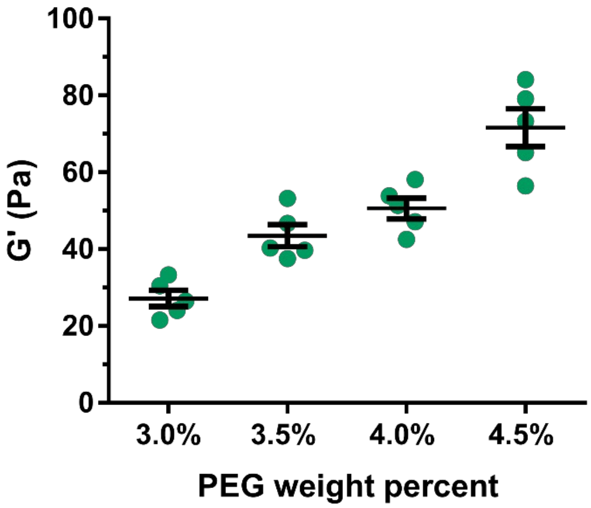

Supplementary Fig. 5. Hydrogel storage modulus is tunable by adjusting polymer density. Storage modulus of bulk hydrogels functionalized with 1.0 mM GFOGER determined by rheological measurements. n = 5 biologically independent samples; mean ± SE. Linear regression: G' = 28.1 PEG density – 57.2, $R^2 = 0.968$, $p < 0.0159$.

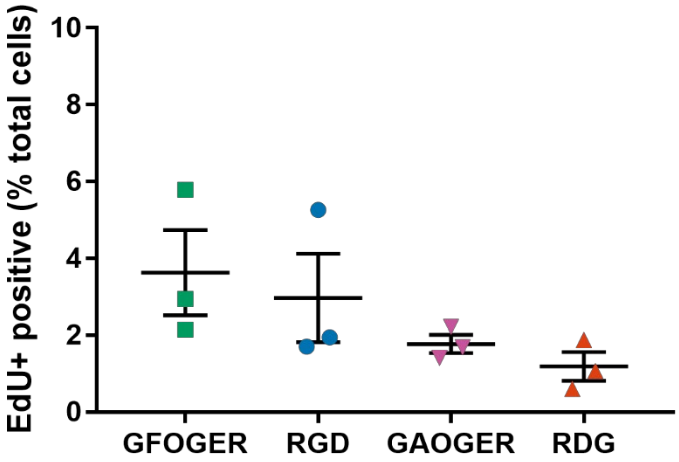

Supplementary Fig. 6. Proliferation of encapsulated hMSC in peptide-functionalized hydrogels. Proliferation was assessed by EdU incorporation (48 h exposure) after 4 days in culture of encapsulated hMSC. No significant differences were detected among peptides by ANOVA ($p = 0.2246$). $n = 3$ biologically independent gels reflecting the average of 4 images/gel; mean ± SE.

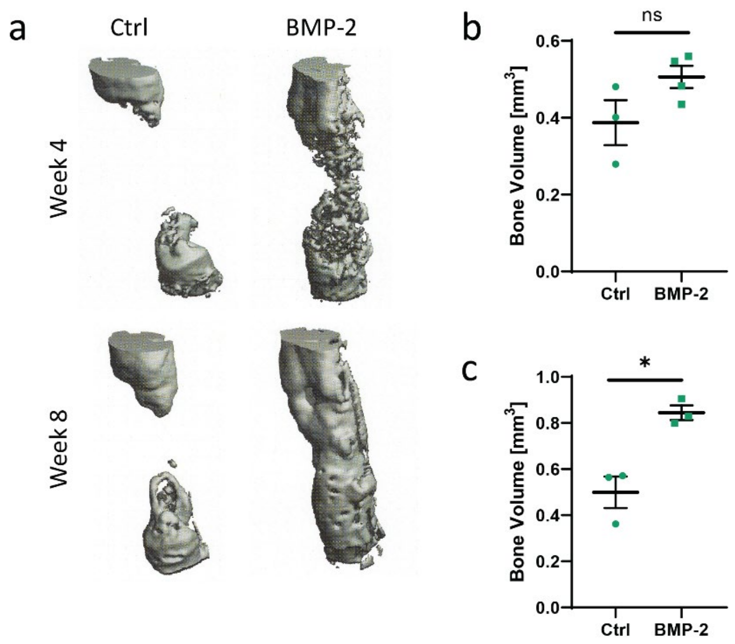

Supplementary Fig. 7. Radial segmental defect is a non-healing defect in NSG mice but heals in response to therapeutic doses of BMP-2. Segmental defects were treated with GFOGER-presenting PEG hydrogel containing BMP-2 (50 ng) or saline (Ctrl). (a) Representative 3-D μCT reconstructions for 4 and 8 weeks post-treatment. (b) Week 4 and (c) week 8 bone volume measurements obtained by μCT. Each point represents a biologically independent sample (BMP-2 = 4 mice (4 weeks), 3 mice (8 weeks), Ctrl = 3 mice (4 weeks), 3 mice (8 weeks)), mean ± SE. Unpaired t-test was used to detect differences (*$p < 0.05$).

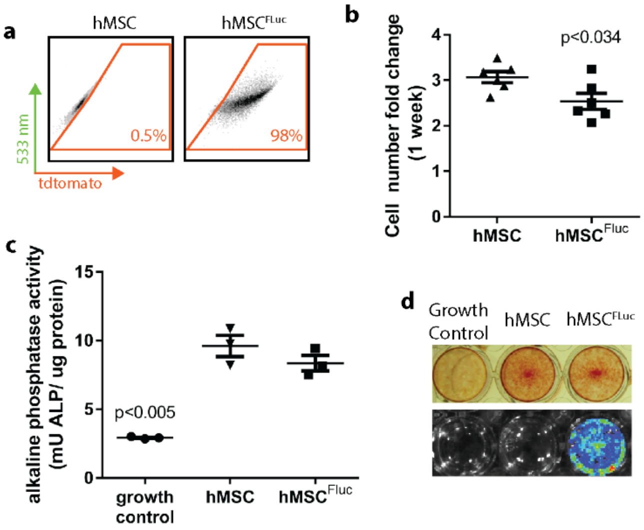

Supplementary Fig. 8. hMSC^{FLuc} exhibit normal growth and differentiation capacities. (a) hMSC were transduced at high efficiency (>90%) with a lentivirus to co-express red firefly luciferase and tdtomato under the ubiquitin C promoter. (b) Fold change in cell number by DNA content over 1 week for unmodified hMSC or hMSC^{FLuc}. N = 6 biological replicates per group, mean ± SE. Two-tailed t-test was used to detect significant differences. (c) ALP activity and (d) mineral deposition by Alizarin red staining (top panel) in response to osteogenic stimulation and hMSC^{FLuc} continued to express luciferase after differentiation (bottom panel). n = 3 biologically independent samples per group, mean ± SE. ANOVA with Tukey's pairwise comparisons was used to detect significant differences.

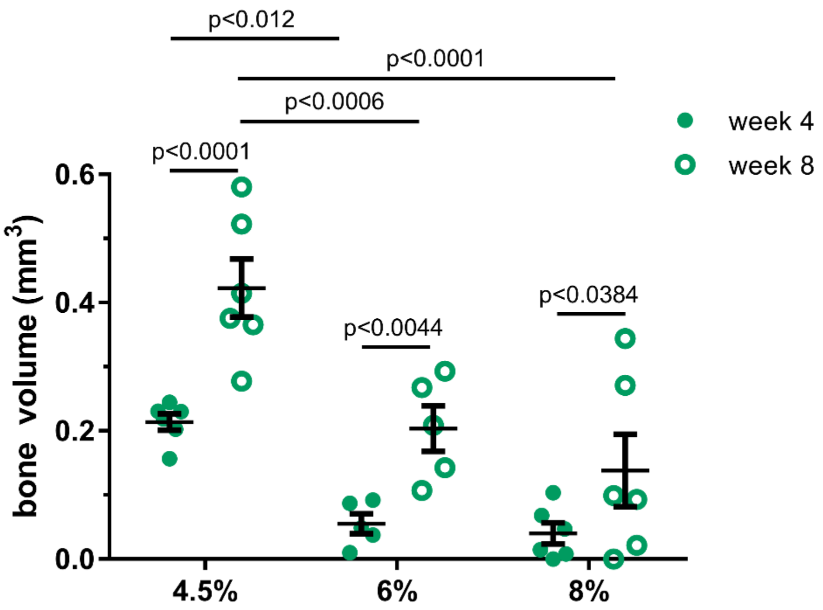

Supplementary Fig. 9. Effect of PEG-4MAL density and hMSC delivery on bone repair. Bone volume of defects treated with 4.5%, 6.0%, or 8.0% PEG-4MAL w/v with 1.0 mM GFOGER and 15,000 hMSC. n = 6 mice per group, mean ± SE. Two-way repeated ANOVA with Tukey's multiple comparisons test (polymer density: $p < 0.0002$, time: $p < 0.0001$).

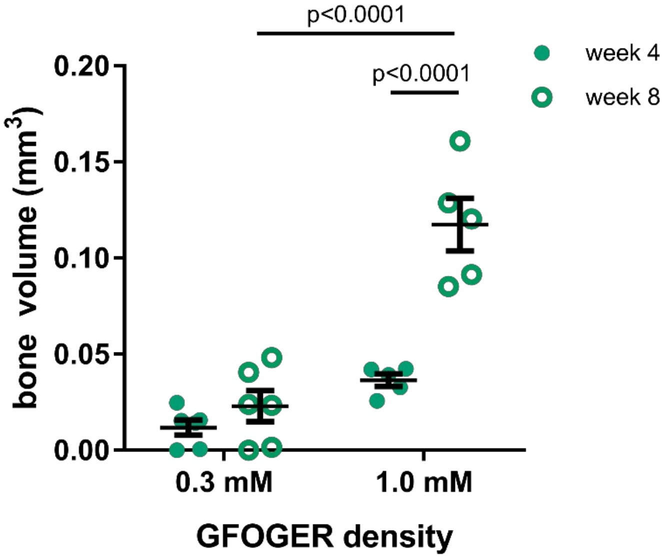

Supplementary Fig. 10. Effect of peptide density on bone repair. Bone volume at weeks 4 and 8 after radial segmental defect treatment with 4.5% PEG hydrogels functionalized with 0.3 mM or 1.0 mM GFOGER without cells. Each point represents a biologically independent sample (0.3 mM = 6 mice, 1.0 mM = 5 mice), mean ± SE. Two-way repeated ANOVA with Tukey's multiple comparisons test (GFOGER density: $p < 0.0001$, time: $p < 0.0001$).

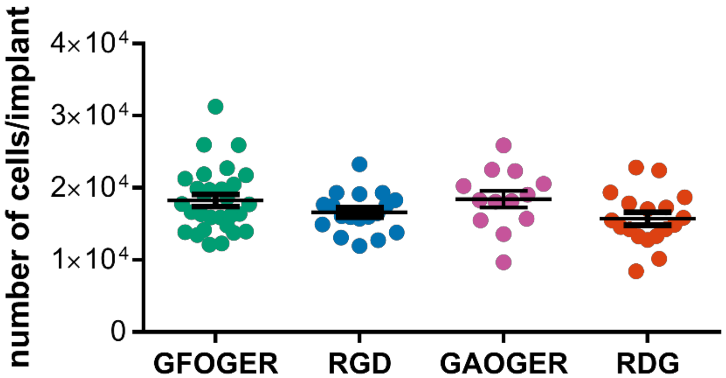

Supplementary Fig. 11. hMSC encapsulation efficiency in hydrogels cast within polyimide sleeves is independent of adhesive peptide type. Encapsulated hMSC number was quantified by DNA content for a sister subset of implants. Each point represents a biologically independent sample (Sample size: GFOGER = 29, RGD = 18, GAOGER = 13, RDG = 18) over 3 independent experiments; mean ± SE. ANOVA showed no differences among groups ($p = 0.11$).

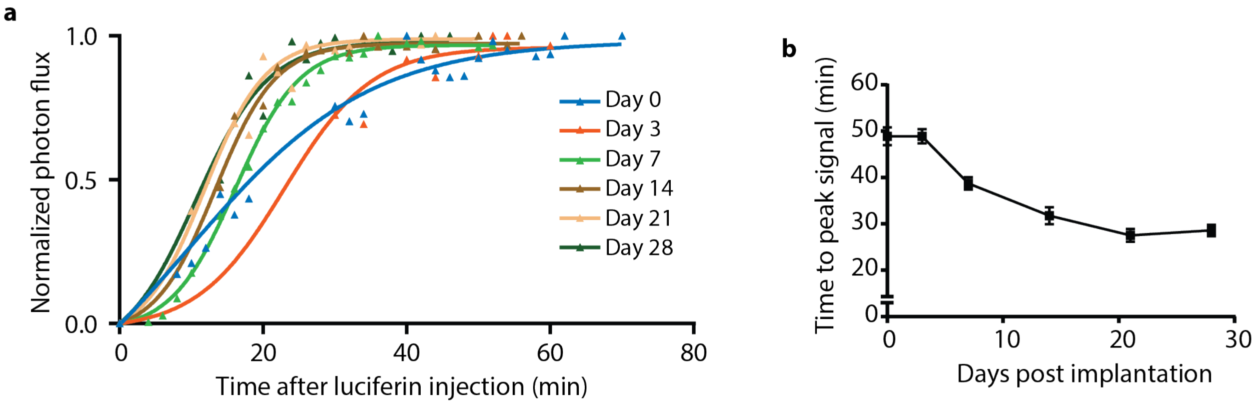

Supplementary Fig. 12. Time to reach peak signal at different implantation time points. (a) Bioluminescence signal was monitored over time after luciferin injection and normalized to the maximum signal for each time point. Data comprises longitudinal bioluminescence readings for 12 mice. (b) Time-to-peak signal decreased with time after surgery until stabilizing after 7 days. Plot shows longitudinal measurements (mean ± SE) for 20 mice.

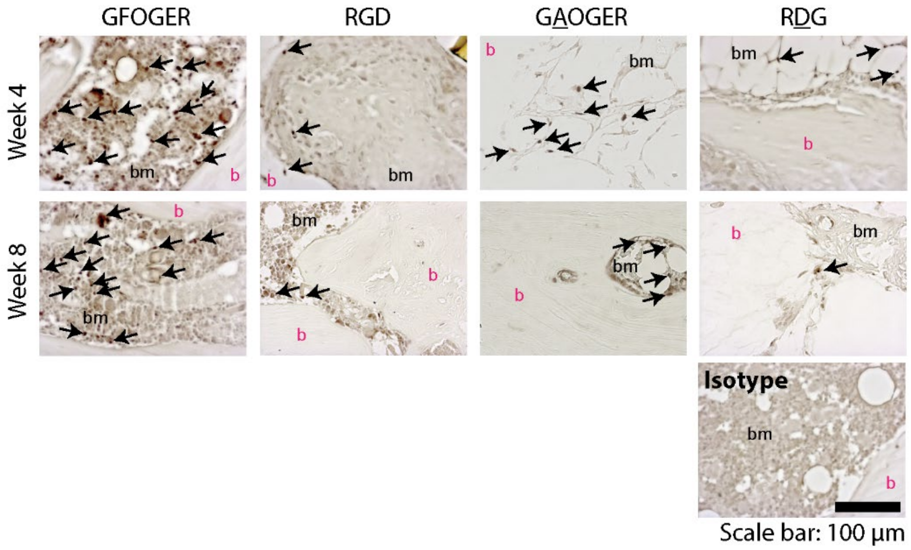

Supplementary Fig. 13. Immunostaining for human-specific nuclear mitotic antigen (NuMa) in radial defects at week 8. Black arrows denote NuMA-positive nuclei (b: bone, bm: bone marrow).

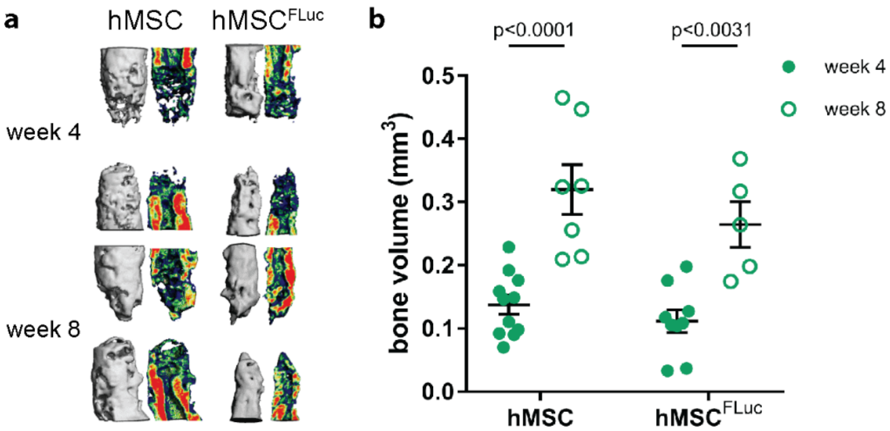

Supplementary Fig. 14. Lentiviral transduction to express luciferase does not alter bone repair by hMSC. (a) Representative 3-D µCT reconstructions and (b) bone volume at weeks 4 and 8 after 15,000 hMSC or hMSC^{FLuc} delivery to the radial segmental defect in 4.5% PEG hydrogels with 1.0 mM GFOGER. Each point represents a biologically independent sample (hMSC = 11 mice (4 weeks), 7 mice (8 weeks), hMSC^{Fluc} = 9 mice (4 weeks), 5 mice (8 weeks)), mean ± SE. Two-way ANOVA with Tukey's multiple comparisons test (time: $p < 0.0001$; cell type: not significant, $p = 0.130$).

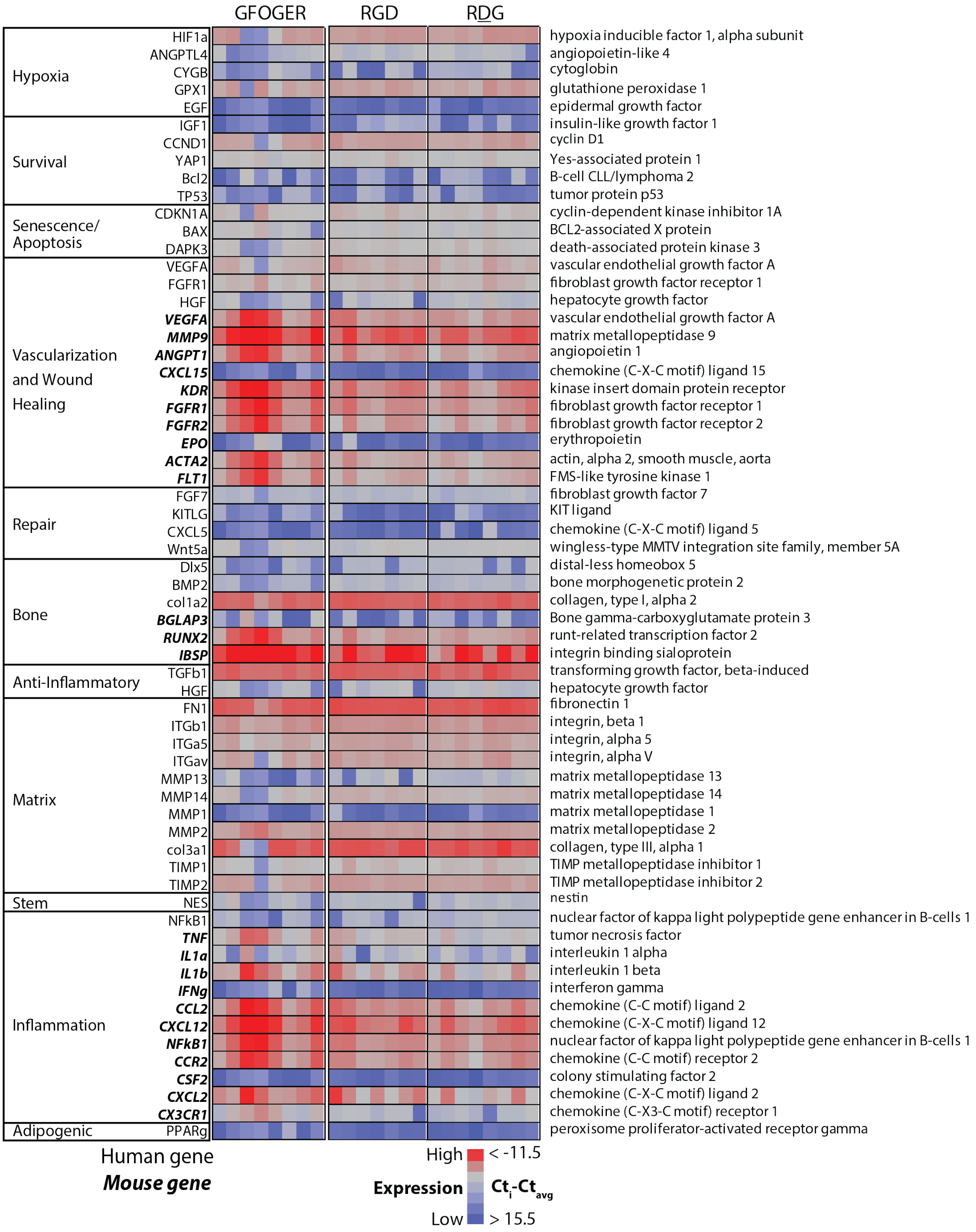

Supplementary Fig. 15. Gene expression heat map. hMSC transplantation in GFOGER-presenting hydrogels resulted in upregulation of inflammation, vascularization and bone genes *in vivo* after 1 week

compared to RGD- and RDG-functionalized gels. Tissue was explanted from the defect after 1 week and total RNA was extracted and standardized across all samples. 96 total genes were screened using gene chip technology resulting in differential expression of ~60 genes. Mean normalized Ct values were used for the analysis and are represented in the heat map where red is high expression and blue is low expression.

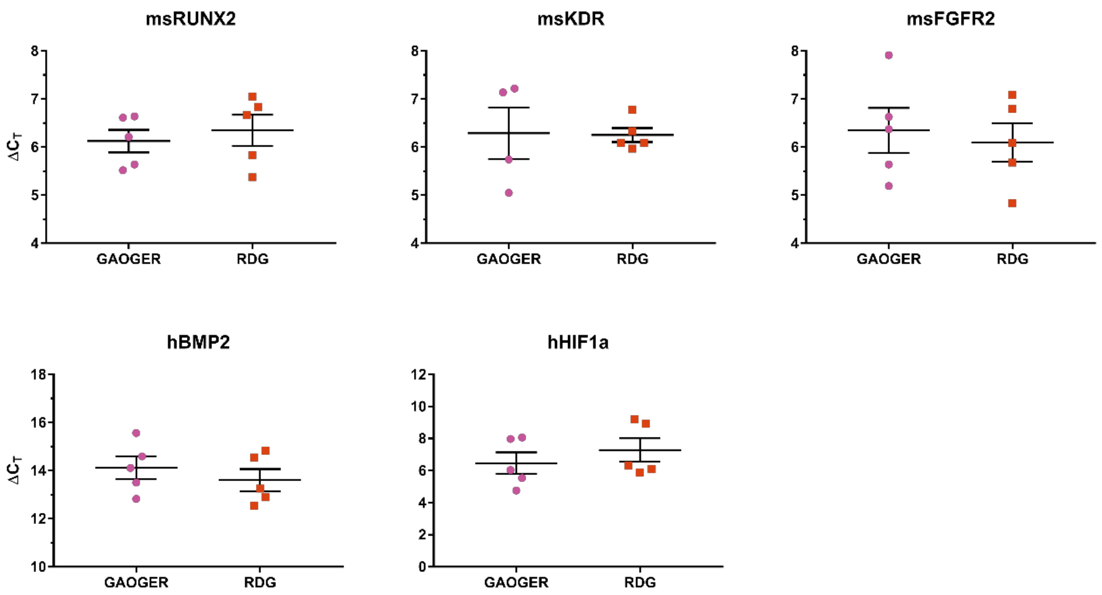

Supplementary Fig. 16. qRT-PCR for selected genes for hMSC-laden hydrogels implanted in segmental defect at 1 week. n = 5 mice per group, mean ± SE. ANOVA showed no differences in gene expression between groups.

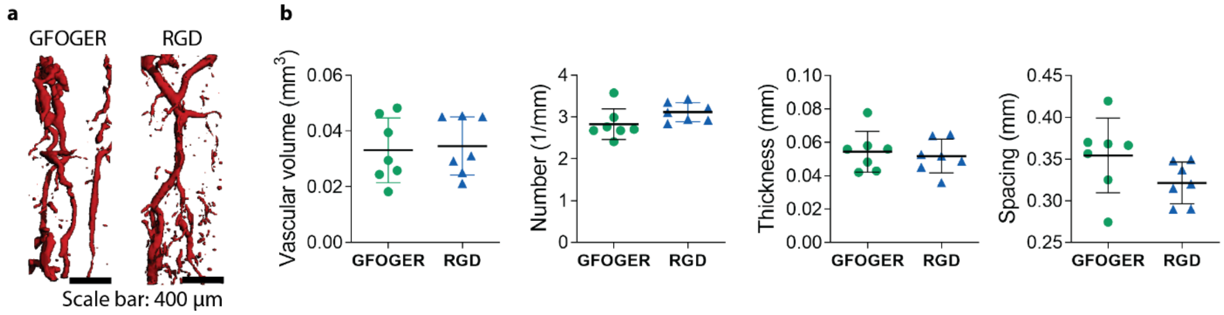

Supplementary Fig. 17. Effect of adhesive peptide on vascularization in the defect. (a) Representative 3D reconstructions of vascular structures within the bone defect at week 8. (b) Vascular volume, number, thickness and spacing in bone defect respectively. Mean ± SD, n = 7-8 mice per group; two-tailed t-test: $^{*}p < 0.05$.

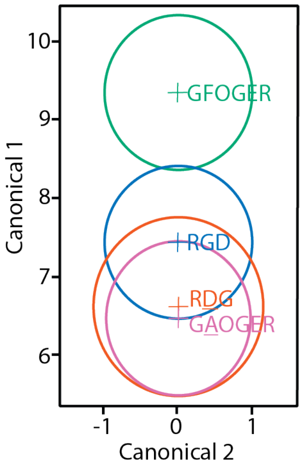

Supplementary Fig. 18. hMSC in GFOGER-presenting hydrogels exhibit differential secretory cytokine profile compared to other hydrogels. Multivariate ANOVA with a sum combination across cytokines showed that hMSC encapsulated in GFOGER-functionalized hydrogels exhibit significantly different cytokine profile under growth conditions. n = 4 biologically independent samples; MANOVA: $p < 0.001$. Multivariate ANOVA was used to detect significant differences. "+" symbol corresponds to each multivariate mean, and ellipses represent a 95% confidence level.

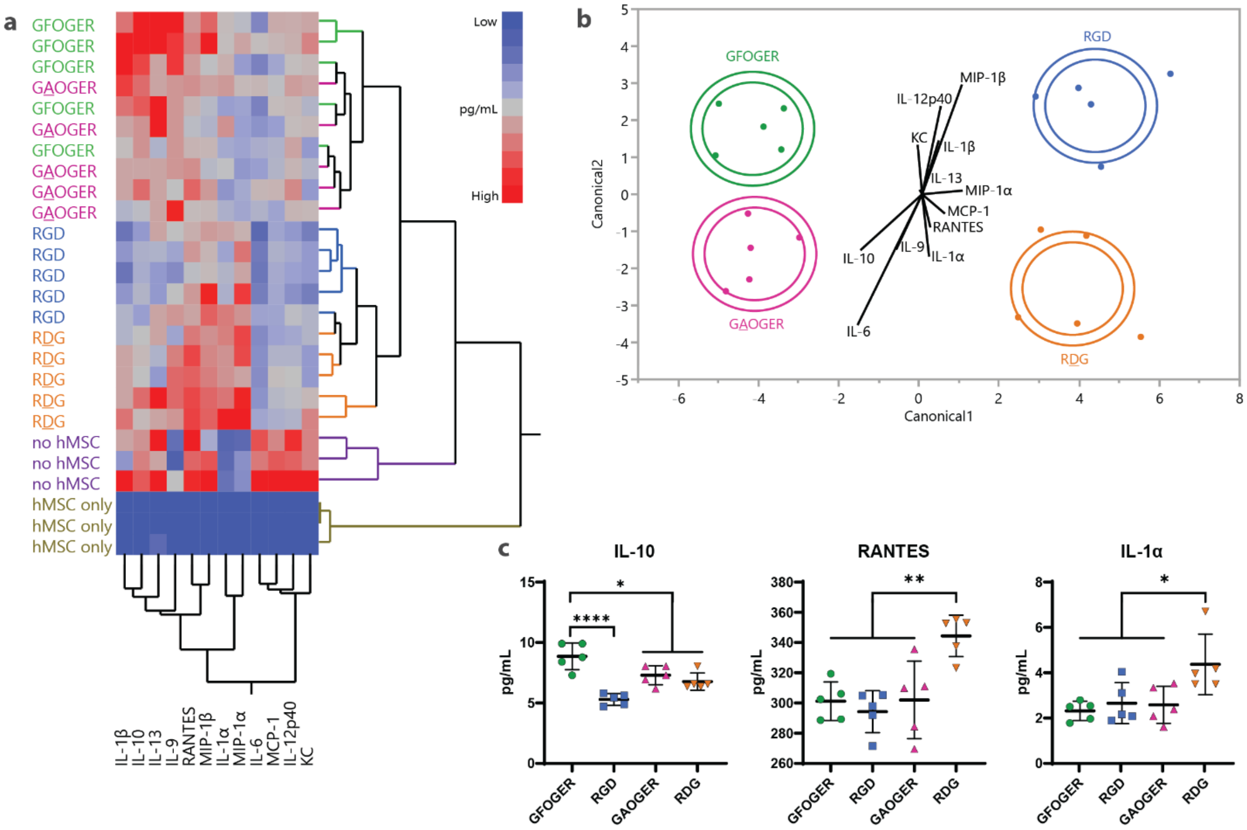

Supplementary Fig. 19. Conditioned media from hMSC cultured within adhesive peptide-presenting hydrogels modulates cytokine secretion from murine macrophages. hMSC-laden hydrogels were co-cultured in transwells with IFN-γ- and LPS-stimulated M1 murine macrophages for 3 days. Conditioned media from the co-cultures was analyzed for macrophage-secreted mouse cytokines using Luminex bead-based multiplex technology. (a) Hierarchical clustering using Ward's method shows clustering of cytokine profiles by adhesive peptide. (b) Linear discriminant analysis of macrophage secreted cytokines. Each point represents a sample and each multivariate mean is a labeled circle corresponding to a 95% confidence limit for the mean. Groups that are significantly different have non-intersecting circles. (c) Cytokines from the multiplex array exhibiting significantly different secretion levels. n = 5 biologically independent samples, mean ± SD; *$p < 0.05$, **$p < 0.01$, ****$p < 0.0001$. ANOVA was used to detect statistical differences followed by Tukey's multiple comparisons test with adjustment for multiple comparisons.

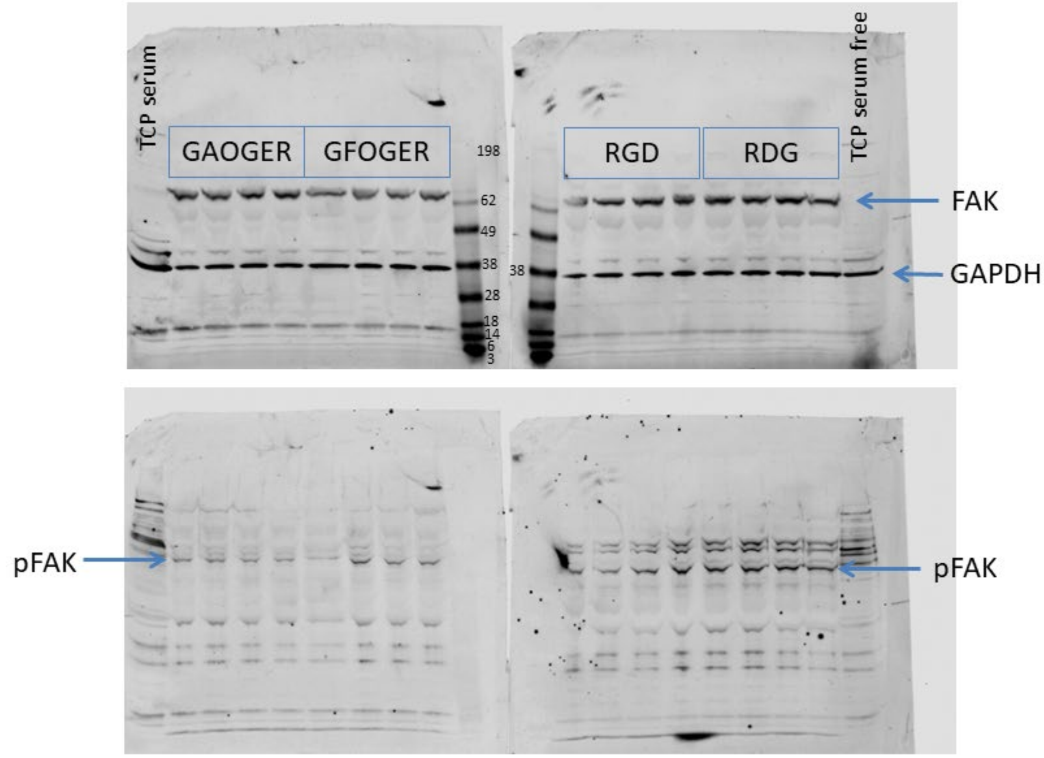

Supplementary Fig. 20. Western blots for FAK and phosphoY397 FAK for hMSC encapsulated in hydrogels presenting integrin-specific peptides. Cell lysates from 4 independent biological samples were separated by SDS-PAGE and transferred to PVDF members. Membranes were probed with mouse monoclonal antibody against GAPDH (Abcam), mouse monoclonal antibody against FAK and rabbit polyclonal antibody against FAK [pY397] (ThermoFisher) at a 1:1000 dilution in 5% BSA TBS-T solution followed by fluorescent (green, red) secondary antibodies (Li-Cor). Immunoblots were visualized on a Li-Cor Odyssey imaging system and grayscale images were analyzed using Image Studio Lite (Li-Cor).

Supplementary Table 1. Mesh size calculations for a 4.5% (w/v) PEG hydrogel with 1.0 mM adhesive peptide.

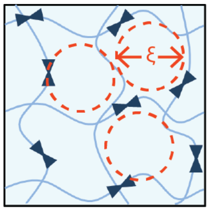

Ligand	ξ ± SD (nm)[a]	ξ ± SD (nm)[b]	ξ ± SD (nm)[c]
GFOGER	40 ± 0.7	18 ± 0.7	23 ± 0.5
RGD	40 ± 1.4	17 ± 0.8	22 ± 0.5
RDG	41 ± 1.0	-	-
GAOGER	41 ± 2.0	-	-

[a] Calculated by rubber elasticity theory and rheological measurements
[b] Calculated by Flory and Rehner equations with experimental M_c
[c] Calculated by Flory and Rehner equations with theoretical M_c

Supplementary Table 2. Secreted cytokine levels of hMSC encapsulated in peptide-functionalized hydrogels. Encapsulated hMSC were maintained in growth medium and medium was conditioned for 48 h and analyzed. Table below shows average cytokine level across ligands. One-way ANOVA: *$p < 0.05$ differences in cytokine level among ligands, n = 4 biologically independent samples per group.

**Cytokine**	**Supernatant Concentration (pg/mL)**
Basic FGF	57.5 ± 5.3*
Eotaxin	ND
G-CSF	ND
GM-CSF	24.8 ± 8.1
IFN-g	630.9 ± 280.5*
IL-1b	3.8 ± 0.6
IL-1ra	65.5 ± 2.8*
IL-2	7.3 ± 7.6
IL-4	2.4 ± 0.4
IL-5	ND
IL-6	1356.8 ± 175.2*
IL-7	5.5 ± 1.1
IL-8	12 ± 3.3*
IL-9	19.1 ± 3.6*
IL-10	65.4 ± 16.3
IL-12p70	98.2 ± 11.8
IL-13	3.1 ± 0.6
IL-15	ND
IL-17a	18.8 ± 4.4
IP-10	19.4 ± 1.5
MCP-1	213.3 ± 46.7*
MIP-1a	1.7 ± 0.1
MIP-1b	4.5 ± 1.1
PDGF-bb	0.4 ± 0.4
RANTES	5.1 ± 3
TNF-a	15.8 ± 7.2
VEGF	5550.2 ± 1300.6*

Supplementary Table 3. Antibodies used for integrin profiling, hMSC marker flow cytometry and blocking studies.

Integrin Flow Cytometry

	Target	Vendor	Catalog #	Dilution	Host	Reactivity
mouse	alpha 1	BioLegend	142601	1:100	Armenian Hamster	Mouse
	alpha 2	BioLegend	103501	1:100	Armenian Hamster	Mouse
	alpha 3	R&D	AF2787	1:100	Goat	Mouse
	alpha 4	BD Pharmingen	553154	1:100	Rat	Mouse
	alpha 5	BD Pharmingen	553319	1:100	Rat	Mouse
	alpha 6	EMD Millipore	MAB1982	1:100	Rat	Mouse
	alpha V	BD Pharmingen	550024	1:100	Rat	Mouse
	beta 3	BD Pharmingen	553344	1:100	Armenian Hamster	Mouse
	beta 1	BD Pharmingen	553837	1:100	Armenian Hamster	Mouse
human	alpha 1	R&D	AF5676	1:100	Sheep	Human
	alpha 2	EMD Millipore	MAB1950Z	1:100	Mouse	Human
	alpha 3	R&D	MAB1345	1:100	Mouse	Human
	alpha 4	EMD Millipore	MAB16983	1:100	Mouse	Human/Primate
	alpha 5	DSHB @ UIowa	BIIG2	1:20	Rat	Human/Pig/Rabbit
	alpha 6	R&D	MAB1350	1:100	Mouse	Human
	alpha V	EMD Millipore	MAB2021Z	1:20	Mouse	Human
	alphaVbeta3	EMD Millipore	MAB1976Z	1:100	Mouse	Human
	beta 1	EMD Millipore	MAB1951Z	1:100	Mouse	Human
	beta 1	DSHB @ UIowa	AIIB2	1:20	Rat	Human/Pig/Dog/Mouse/Sheep
	beta 3	R&D	AF2266	1:100	Goat	Human
isotype	IgG1 Isotype	EMD Millipore	CBL610	1:100	Mouse	
	IgG Isotype	BioLegend	400901	1:100	Armenian Hamster	
	IgG Isotype	R&D	AB-108-C	1:100	Goat	
	IgG Isotype	R&D	MAB005	1:100	Rat	
	IgG Isotype	Vector Labs	I-1000	1:100	Rabbit	
	IgG Isotype	Thermo	31243	1:100	Sheep	

hMSC Marker Flow Cytometry

Target	Vendor	Catalog #	Dilution	Host	Reactivity
FITC anti-human CD14	Biolegend	367116	1.5:300	Mouse	Human
FITC anti-human CD34	Biolegend	343504	1.5:300	Mouse	Human
FITC anti-human CD45	Biolegend	368508	1.5:300	Mouse	Human
FITC anti-human CD73 (Ecto-5'-nucleotidase)	Biolegend	344016	1.5:300	Mouse	Human
FITC anti-human CD90 (Thy1)	Biolegend	328108	1.5:300	Mouse	Human
FITC anti-human CD105	Biolegend	323204	1.5:300	Mouse	Human
FITC Mouse IgG1, κ Isotype Ctrl	Biolegend	400110	1.5:300	Mouse	

Blocking

Target	Vendor	Catalog #	Dilution	Host	Reactivity
alpha 2	EMD Millipore	MAB1950Z	1:100	Mouse	Human
alpha V	EMD Millipore	MAB2021Z	1:20	Mouse	Human
alphaVbeta3	EMD Millipore	MAB1976Z	1:100	Mouse	Human
beta 1	DSHB @ UIowa	AIIB2	1:20	Rat	Human/Pig/Dog/Mouse/Sheep
IgG1 isotype	EMD Millipore	CBL610	1:100	Mouse	

Western blot
GAPDH (Abcam, ab9485, rabbit, reactive against human, mouse, rat, chicken, others)
FAK (ThermoFisher, 39-6500, mouse, reactive against human, mouse, rat)
FAK [pY397] (ThermoFisher, 44-624G, rabbit, reactive against human, mouse, rat, Xenopus, others)

Immunostaining
NuMa (Abcam, ab84680, rabbit, reactive against human, marmoset)
ImmPRESS HRP anti-rabbit IgG (Vector Labs, MP-7401-15, horse, reactive against rabbit)

Supplementary Table 4. Primers (ThermoFisher) used for gene expression studies.

Assay ID (Hs (human), Mm (mouse))	Gene Target	Catalog Number
Hs01037003_g1	MMP14	4448892
Hs01552918_m1	FGFR2	4448892
Mm04208136_m1	Cxcl15	4448892
Mm01202755_m1	Epo	4448892
Mm00649782_gH	Bglap3	4448892
Hs01028969_m1	COL1A2	4448892
Hs01001343_g1	SOX9	4448892
Hs03676628_s1	BMP4	4448892
Hs01115610_m1	PTGES	4448892
Hs01092738_m1	FGF1	4448892
Hs00902712_g1	YAP1	4448892
Hs00794094_m1	TAZ	4448892
Hs00370478_m1	CYGB	4448892
Hs00154676_m1	DAPK3	4448892
Hs00205071_m1	GNL3	4448892
Hs04187831_g1	NES	4448892
Hs01060665_g1	ACTB	4448892
Hs01099660_g1	CXCL5	4448892
Hs01573504_m1	WNT4	4448892
Mm00492555_m1	Ibsp	4453320
Hs01029057_m1	MMP8	4453320
Hs00932747_m1	TGFBI	4453320
Hs01931883_s1	GDNF	4453320
Hs00940253_m1	FGF7	4453320
Hs01866874_s1	SP7	4453320
Mm01216173_m1	Ccr2	4453320
Hs00193291_m1	DLX5	4453320
Hs00300159_m1	HGF	4453320
Hs00300159_m1	HGF	4453320
Hs01587814_g1	BGLAP	4453320
Mm02620111_s1	Cx3cr1	4453320
Hs00233808_m1	ITGAV	4453320
Hs00829989_gH	GPX1	4453320
Hs00269972_s1	CEBPA	4453320
Mm00438980_m1	Flt1	4453320
Hs00559595_m1	ITGB1	4453320
Mm00456503_m1	Angpt1	4453320
Hs00234278_m1	TIMP2	4453320
Hs00915142_m1	FGFR1	4453320
Hs00966522_m1	PDGFB	4453320

Mm01290062_m1	Csf2	4453320
Hs00241497_m1	KITLG	4453320
Hs00154192_m1	BMP2	4453320
Hs00233476_m1	BMP7	4453320
Hs00765730_m1	NFKB1	4453320
Hs01547673_m1	ITGA5	4453320
Mm00436450_m1	Cxcl2	4453320
Hs00899658_m1	MMP1	4453320
Hs00171558_m1	TIMP1	4453320
Hs00365052_m1	FN1	4453320
Hs00943809_m1	COL3A1	4453320
Hs00998537_m1	WNT5A	4453320
Hs00231692_m1	RUNX2	4453320
Mm00725412_s1	Acta2	4453320
Mm01269930_m1	Fgfr2	4453320
Hs01055668_m1	LIF	4453320
Hs00608023_m1	BCL2	4453320
Hs00607978_s1	CXCR4	4453320
Hs01547656_m1	IGF1	4453320
Hs01101127_m1	ANGPTL4	4453320
Hs00181225_m1	FASLG	4453320
Hs00153153_m1	HIF1A	4453320
Mm00442991_m1	Mmp9	4453320
Mm01222421_m1	Kdr	4453320
Hs01099999_m1	EGF	4453320
Hs00180269_m1	BAX	4453320
Mm00439620_m1	Il1a	4453320
Mm00445553_m1	Cxcl12	4453320
Hs00158127_m1	ITGA2	4453320
Mm01545399_m1	Hprt	4453320
Hs00264051_m1	COL2A1	4453320
Mm00438930_m1	Fgfr1	4453320
Hs00243522_m1	TNFSF11	4453320
Hs00961622_m1	IL10	4453320
Hs00234579_m1	MMP9	4453320
Hs00234140_m1	CCL2	4453320
Hs01548727_m1	MMP2	4453320
Mm00434228_m1	Il1b	4453320
Hs01075529_m1	NOS2	4453320
Hs00355782_m1	CDKN1A	4453320
Mm01168134_m1	Ifng	4453320
Hs00900055_m1	VEGFA	4453320
Hs00985639_m1	IL6	4453320

Hs00233992_m1	MMP13	4453320
Hs00984148_m1	IDO1	4453320
Mm00443258_m1	Tnf	4453320
Hs02758991_g1	GAPDH	4453320
Mm00501584_m1	Runx2	4453320
Mm00607939_s1	Actb	4453320
Mm01281449_m1	Vegfa	4453320
Hs01034249_m1	TP53	4453320
Hs00153408_m1	MYC	4453320
Hs01115513_m1	PPARG	4453320
Mm00441242_m1	Ccl2	4453320
Hs00765553_m1	CCND1	4453320
Mm00476361_m1	Nfkb1	4453320
Mm99999915_g1	GAPDH	4331182

SUPPLEMENTARY METHODS

hMSC integrin profiling and surface marker expression

Live cell flow cytometry analysis of hMSCs was performed on a FACSAria III flow cytometer (BD Biosciences). The antibodies and dilutions used for cell staining are listed in **Supplementary Table 3**.

Peptide-PEG-4MAL tethering efficiency

Four-arm, maleimide-end functionalized (>95%) PEG macromer (PEG-mal, 20 kDa, Laysan Bio) was reacted with GFOGER peptide (GGYGGGPG$(GPP)_5$GFOGER$(GPP)_5$GPC, O = hydroxyproline), RGD peptide (GRGDSPC), or the scrambled, non-adhesive RDG peptide (CRDGSPC) in 10 mM HEPES in PBS, pH 7.4 for 15 min at 37 ºC. The reaction diluted in PBS 100-fold and diluted PEG-adhesive peptide (10 μL) plus thiol-quantitation reagent (100 μL, Measure-iT Thiol Assay Kit, Thermo Fisher) was added per well of a 96-well plate and read using a microplate reader. Dilutions of GFOGER, RGD, or RDG in 10 mM HEPES in PBS were used as standards. All samples and standards were measured in triplicate.

Rheometry

12.5 μL hydrogels were cast as discs in 4.5 mm diameter silicone isolators (Grace Bio-Labs, Sigma) on Sigmacote-treated slides (Sigma). The gels were allowed to cross-link at 37 ºC, removed from the isolators, and swollen in PBS overnight. Rheological measurements were made using a cone and plate rheometer (MCR302, Anton Paar). The sample was loaded onto the plate, the cone was lowered, and excess sample was removed. Storage and loss moduli were measured over a range of angular frequencies with a strain that corresponded to the linear viscoelastic region.

Swelling studies

50 μL hydrogels were allowed to cross-link at 37 ºC and swollen in PBS overnight. Swollen hydrogels were weighed and snap frozen in liquid nitrogen. Following lyophilization, dry hydrogels were weighed. Mass swelling ratio is presented as

$$Q = \frac{mass_{wet}}{mass_{dry}} \tag{1}$$

Mesh size calculations

Mesh size was calculated using several methods: 1. rheological measurements and rubber elasticity theory [1], and swelling measurements and the Flory and Rehner equation modified by Merrill and Peppas [2, 3] based on 2. experimental data or 3. theoretical values. Rubber elasticity theory relates mesh size, ξ, to storage modulus, G' as follows:

$$\xi = \left(\frac{G' N_A}{RT}\right)^{-\frac{1}{3}} \tag{2}$$

where R is the molar gas constant and T the temperature. Swelling measurements were used to calculate mesh size using the following equations:

$$\xi = {v_{2,s}}^{-\frac{1}{3}} (\bar{r}_0^2)^{\frac{1}{2}} \tag{3}$$

$$(\bar{r}_0^2)^{\frac{1}{2}} = l \left(\frac{2\bar{M}_c}{M_r}\right)^{\frac{1}{2}} C_n^{\frac{1}{2}} \tag{4}$$

$$\frac{1}{\bar{M}_c} = \frac{2}{\bar{M}_n} - \frac{\left(\frac{\bar{v}}{V_1}\right)\left[ln\left(1 - v_{2,s}\right) + v_{2,s} + \chi {v_{2,s}}^2\right]}{v_{2,r}\left[\left(\frac{v_{2,s}}{v_{2,r}}\right)^{\frac{1}{3}} - \frac{1}{2}\left(\frac{v_{2,s}}{v_{2,r}}\right)\right]} \tag{5}$$

where $v_{2,\mathrm{s}}$ and $v_{2,\mathrm{r}}$ are the polymer volume fraction of the gel in the swollen and relaxed states, respectively, $(\bar{r}_0^2)^{\frac{1}{2}}$ is the unperturbed mean-square end-to-end distance of the PEG, l is the average value of the bond length = 1.46 Å, $\bar{M}_c$is the average molecular mass between the cross-links in the network (experimental value calculated from supplementary equation (5); theoretical value taken as $2 * MW_{multimer\ arm} + MW_{crosslinking\ peptide}$), $M_r$is the molecular mass of PEG repeating unit (44 g/mol), $C_n$is the characteristic

ratio of PEG = 4, $\bar{v}$ is the specific volume of PEG $\bar{v} = \frac{\rho_{H2O}}{\rho_{PEG}} = \frac{1\frac{g}{cm^3}}{1.12\frac{g}{cm^3}}$, $V_1$is the molar volume of the solvent (18 cm^3/mol for water), and χ is the polymer-solvent interaction parameter (0.4 for PEG-water).

EdU staining for cell proliferation

hMSC-laden hydrogels were cultured free-floating in media. At day 7, cells were incubated with 10 μM EdU for 48 hr. EdU detection was visualized using a Click-iT EdU kit according to manufacturer's instructions (Thermo Fisher). Nuclei were stained with DAPI and visualized with a Nikon C2 laser scanning confocal head on a Nikon Eclipse-Ti microscope and Elements software (Nikon) and images were analyzed using ImageJ (NIH).

$hMSC^{FLuc}$ characterization

Cell growth capacity for hMSC transduced to express luciferase ($hMSC^{FLuc}$) compared to hMSC was measured by fold change in DNA content over 7 days. 1000 hMSC or $hMSC^{FLuc}$ were seeded into wells of a 96 well plate and cultured in growth media. At days 1 and 7, cells were rinsed with PBS and plates were stored at -80 ºC until analysis. DNA content was measured using the CyQuant kit (Thermo Fisher) according to the manufacturer's instructions. Differentiation capacity for $hMSC^{FLuc}$ compared to hMSC was measured by alkaline phosphatase activity (ALP) and mineralization. hMSC were seeded at 10,000 cells/cm^2 on tissue culture plastic and cultured in osteogenic differentiation medium (basal media with dexamethasone, ascorbate, mesenchymal cell growth supplement, L-glutamine, penicillin/streptomycin, and sodium β-glycerophosphate, Lonza). After 9 days of culture in osteogenic differentiation medium, cells were lysed and assayed for ALP by incubating with MUP substrate. hMSC were scraped in cold 50 mM Tris-HCl and sonicated to lyse the cells. The total protein content for each lysate sample was determined using a BCA assay kit (Thermo Scientific). Samples and ALP standards were loaded into a 96-well plate, then incubated with 60 μg/mL MUP substrate at 37 ºC for 1 hr and read at 360 nm excitation/465 nm emission. ALP activity was normalized to sample protein content. After 21 days in induction media, luciferase expression and mineralization were assessed. To visualize luciferase expression after differentiation, luciferin (150 μg/mL) was added to the cells and bioluminescence imaged on an IVIS

Lumina II (Perkin Elmer). Mineral deposition was visualized by Alizarin red staining. Cells were fixed in 10% formalin, rinsed in water, incubated in 2% Alizarin red solution for 20 min, and washed 4 times with water.

Implant cell loading

The hydrogel was prepared as previously described and individual implant sleeves were filled. Hydrogels were allowed to crosslink and swell in complete media. Cell loading efficiency was quantified for sister implant samples by DNA content. Implants were incubated in 1.0 mg/mL collagenase, type I (ThermoFisher) at 37 °C until fully degraded. Cells were lysed by sonication and freeze-thaw cycles. Whole cell lysate was assayed for DNA content and cell number using a CyQuant kit according to the manufacturer's instructions (ThermoFisher) and compared to a cell standard curve.

Histology and immunostaining

Animals were euthanized 8 weeks after surgery by CO_2 inhalation and their radii and ulna were harvested. Soft tissue was removed carefully without disturbing the defect and the bones fixed in 10% neutral buffered formalin overnight. Samples were briefly rinsed in tap water and decalcified in formic acid for two days. The samples were processed for paraffin embedding and sectioned to a 5 μm thickness. For human-specific staining, sections were deparaffinized and hydrated and treated with sodium citrate buffer, pH 6.0, at 60 °C overnight for antigen retrieval. Endogenous peroxidase was inhibited with 3% H_2O_2 followed by blocking with 2.5% horse serum for 1 h at room temperature (Vector Labs). Sections were stained with human-specific anti-NuMa (ab84680, Abcam, 1:100) or rabbit IgG isotype control (1 μg/mL, Vector Labs) at 4 °C overnight, followed by ImmPRESS™ HRP Anti-Rabbit IgG (Vector labs) for 1 h at room temperature. The stain was developed with ImmPACT™ DAB (Vector labs) and sections were dehydrated and mounted.

hMSC-macrophage co-culture assay

Primary murine monocytes were isolated from the bone marrow of C57BL/6J mice (5-8 week old, Jackson Lab). Following sacrifice by CO_2 inhalation, the mouse femura and tibiae were isolated and soft tissue was removed. The end of a 200 μL pipet tip was cut off and the pipet tip was placed in a 1.5 mL

Eppendorf tube. The proximal ends of the bones were cut with scissors until the marrow was visible then the bone was inverted into the pipet tip in the Eppendorf tube. The bones were then spun at 10,000*g* for 10 sec to pellet the marrow in the bottom of the Eppendorf tube. The bones were discarded and the marrow was resuspended in DPBS with 10% FBS and transferred to a flow cytometry tube. Monocytes were isolated from the whole bone marrow using the MACS Monocyte Isolation Kit (BM), mouse (Miltenyi Biotec, 130-100-629) and LS columns (Miltenyi Biotec, 130-042-401).

Isolated primary murine monocytes were seeded in a 6-well non-tissue culture plastic plate at a density of 50,000 cells/cm^2. Cells were matured to macrophages for 7 days in RPMI 1640 media (Gibco, 11875-085) with 10% heat-inactivated fetal bovine serum, 1% pen/strep, and 20 ng/mL murine M-CSF (Biolegend, 574804). On day 7, macrophages were fed with media containing 20 ng/mL M-CSF (Biolegend 574804), 50 ng/mL IFN-γ (Biolegend 570204), and 100 ng/mL LPS (Invitrogen 00-4976-93) and allowed to polarize towards the M1 macrophage phenotype for 24 h.

hMSC-laden hydrogels were made 48 h prior to co-culture. On day 8 of macrophage culture, the M1 polarizing media was removed and the cells were gently washed with PBS. The hMSC-laden hydrogels were placed in a Transwell (3 μm pore, Corning, 3414) and added to the well above the macrophages. The co-culture were fed with 2 mL of base RPMI media and 2 mL of hMSC conditioned αMEM (conditioned during the first 48 h of hMSC culture in the gels). The hMSC-free control was fed with 2 mL of RPMI and 2 mL of αMEM containing 20 ng/mL M-CSF.

After 72 h, conditioned media was removed from the co-culture and prepared for Luminex assay as described above. Murine macrophage cytokine secretions were then analyzed using the Bio-Plex Pro Mouse Cytokine 23-plex Assay (Bio-Rad, m60009rdpd).

References

1. Welzel, P.B. et al. Modulating Biofunctional starPEG Heparin Hydrogels by Varying Size and Ratio of the Constituents. *Polymers* **3**, 602-620 (2011).

2. Canal, T. & Peppas, N.A. Correlation between mesh size and equilibrium degree of swelling of polymeric networks. *J Biomed Mater Res* **23**, 1183-1193 (1989).

3. Raeber, G.P., Lutolf, M.P. & Hubbell, J.A. Molecularly engineered PEG hydrogels: a novel model system for proteolytically mediated cell migration. *Biophys J* **89**, 1374-1388 (2005).
