# Supplementary material for: Integrin-specific hydrogels modulate transplanted human bone marrow-derived mesenchymal stem cell survival, engraftment, and reparative activities
Source: Nat Commun. 2020 Jan 8;11:114. doi: 10.1038/s41467-019-14000-9 (PMC6949269; doi:10.1038/s41467-019-14000-9)
Supplement: Supplementary file 2 — Reporting Summary [file 41467_2019_14000_MOESM2_ESM.pdf]

nature research

Corresponding author(s): Garcia, Andres

Last updated by author(s): Oct 15, 2019

Reporting Summary

Nature Research wishes to improve the reproducibility of the work that we publish. This form provides structure for consistency and transparency in reporting. For further information on Nature Research policies, seeAuthors & Referees and theEditorial Policy Checklist .

Statistics

For all statistical analyses, confirm that the following items are present in the figure legend, table legend, main text, or Methods section.

n/a	Confirmed	
☐	☒	The exact sample size (*n*) for each experimental group/condition, given as a discrete number and unit of measurement
☐	☒	A statement on whether measurements were taken from distinct samples or whether the same sample was measured repeatedly
☐	☒	The statistical test(s) used AND whether they are one- or two-sided<br>*Only common tests should be described solely by name; describe more complex techniques in the Methods section.*
☐	☒	A description of all covariates tested
☐	☒	A description of any assumptions or corrections, such as tests of normality and adjustment for multiple comparisons
☐	☒	A full description of the statistical parameters including central tendency (e.g. means) or other basic estimates (e.g. regression coefficient) AND variation (e.g. standard deviation) or associated estimates of uncertainty (e.g. confidence intervals)
☐	☒	For null hypothesis testing, the test statistic (e.g. *F*, *t*, *r*) with confidence intervals, effect sizes, degrees of freedom and *P* value noted<br>*Give P values as exact values whenever suitable.*
☒	☐	For Bayesian analysis, information on the choice of priors and Markov chain Monte Carlo settings
☐	☒	For hierarchical and complex designs, identification of the appropriate level for tests and full reporting of outcomes
☒	☐	Estimates of effect sizes (e.g. Cohen's *d*, Pearson's *r*), indicating how they were calculated

Our web collection on statistics for biologists contains articles on many of the points above.

Software and code

Policy information about availability of computer code

Data collection	Adherent cells for the spinning disk assay were counted using a custom macro in Image-Pro analysis software<br>Image acquisition of immunofluorescence experiments performed using Elements software (Nikon)<br>IVIS data was collected using Living Image software (Perkin Elmer)<br>Micro CT data was collected using VivaCT software (Scanco Medical)
Data analysis	Western blot was analyzed using Image Studio Lite (Li-Cor)<br>Spinning disk analysis was done using custom Matlab code<br>Immunofluorescent and Alizarin red images were analyzed using ImageJ<br>IVIS data was analyzed using Living Image software (Perkin Elmer)<br>Micro CT bone volume and mineral density data was analyzed using VivaCT software (Scanco Medical)<br>qPCR microarray and Luminex arrays were analyzed using JMP-Genomics (SAS Institute)<br>All statistical analysis was performed using GraphPad Prism 8

For manuscripts utilizing custom algorithms or software that are central to the research but not yet described in published literature, software must be made available to editors/reviewers. We strongly encourage code deposition in a community repository (e.g. GitHub). See the Nature Research guidelines for submitting code & software for further information.

Data

Policy information about availability of data

All manuscripts must include a data availability statement. This statement should provide the following information, where applicable:

- Accession codes, unique identifiers, or web links for publicly available datasets
- A list of figures that have associated raw data
- A description of any restrictions on data availability

Provide your data availability statement here.

Field-specific reporting

Please select the one below that is the best fit for your research. If you are not sure, read the appropriate sections before making your selection.

[x] Life sciences [ ] Behavioural & social sciences [ ] Ecological, evolutionary & environmental sciences

For a reference copy of the document with all sections, see nature.com/documents/nr-reporting-summary-flat.pdf

Life sciences study design

All studies must disclose on these points even when the disclosure is negative.

Sample size	Sample sizes were selected based on statistical power calculations and previous experience with these metrics.
Data exclusions	Animals that lost 10% of pre-operative weight or developed infections were euthanized and excluded from analysis.
Replication	All experiments were performed on biologically independent samples (gels, mice) in several independent experiments. Results were consistent across independent experimental runs.
Randomization	Animals were randomized among control and treatment groups keeping the initial average weight at similar level for all groups. The surgical order was also randomized.
Blinding	All measurements were electronically and time-stamped recorded. In several instances, measurements were obtained by different users.

Reporting for specific materials, systems and methods

We require information from authors about some types of materials, experimental systems and methods used in many studies. Here, indicate whether each material, system or method listed is relevant to your study. If you are not sure if a list item applies to your research, read the appropriate section before selecting a response.

Materials & experimental systems

n/a	Involved in the study	
[ ]	[x]	Antibodies
[ ]	[x]	Eukaryotic cell lines
[x]	[ ]	Palaeontology
[ ]	[x]	Animals and other organisms
[x]	[ ]	Human research participants
[x]	[ ]	Clinical data

Methods

n/a	Involved in the study	
[x]	[ ]	ChIP-seq
[ ]	[x]	Flow cytometry
[x]	[ ]	MRI-based neuroimaging

Antibodies

Antibodies used

Integrin antibodies:
alpha 1 (BioLegend, 142601)
mouse alpha 2 (BioLegend, 103501)
mouse alpha 3 (R&D, AF2787)
mouse alpha 4 (BD Pharmingen, 553154)
mouse alpha 5 (BD Pharmingen, 553319)
mouse alpha 6 (EMD Millipore, MAB1982)
mouse alpha V (BD Pharmingen, 550024)
mouse beta 3 (BD Pharmingen, 553344)
mouse beta 1 (BD Pharmingen, 553837)
human alpha 1 (R&D, AF5676)

	human alpha 1 (EMD Millipore, MAB1973)<br>human alpha 2 (EMD Millipore, MAB1950Z)<br>human alpha 3 (R&D, MAB1345)<br>human alpha 4 (EMD Millipore, MAB16983)<br>human alpha 5 BIIG2 (DSHB @ U Iowa)<br>human alpha 6 (R&D, MAB1350)<br>human alpha V (EMD Millipore, MAB2021Z)<br>human alphaVbeta3 (EMD Millipore, MAB1976Z)<br>human beta 1 (EMD Millipore, MAB1951Z)<br>human beta 1 AIIB2 (DSHB @ U Iowa)<br>human beta 3 (R&D, AF2266)<br><br>Isotype controls:<br>IgG1 Isotype Ctrl (EMD Millipore, CBL610)<br>IgG Isotype Ctrl (BioLegend, 400901)<br>IgG Isotype Ctrl (R&D, AB-108-C)<br>IgG Isotype Ctrl (R&D, MAB005)<br>IgG Isotype Ctrl (Vector Labs, I-1000)<br>IgG Isotype Ctrl (Thermo, 31243)<br><br>hMSC marker antibodies:<br>FITC anti-human CD14 (Biolegend, 367116)<br>FITC anti-human CD34 (Biolegend, 343504)<br>FITC anti-human CD45 (Biolegend, 368508)<br>FITC anti-human CD73 (Ecto-5'-nucleotidase) (Biolegend, 344016)<br>FITC anti-human CD90 (Thy1) (Biolegend, 328108)<br>FITC anti-human CD105 (Biolegend, 323204)<br>FITC Mouse IgG1, κ Isotype Ctrl (Biolegend, 400110)<br><br>Western blot antibodies:<br>GAPDH (Abcam, ab9485)<br>FAK (ThermoFisher, 39-6500)<br>FAK [pY397] (ThermoFisher, 44-624G)<br><br>Histology antibodies:<br>human NuMa (Abcam, ab84680)<br>ImmPRESS HRP anti-rabbit IgG (Vector Labs, MP-7401-15)
Validation	All antibodies were used for applications and species validated by the manufacturer

Eukaryotic cell lines

Policy information about cell lines

Cell line source(s)	hMSCs were isolated from human donor bone marrow by the NIH at Texas A&M University
Authentication	The NIH at Texas A&M University provides full characterization of the cells with the shipment. We confirmed the cells were hMSCs using flow cytometry for surface marker expression.
Mycoplasma contamination	*Confirm that all cell lines tested negative for mycoplasma contamination OR describe the results of the testing for mycoplasma contamination OR declare that the cell lines were not tested for mycoplasma contamination.*
Commonly misidentified lines<br>(See ICLAC register)	No cells used in this study were found in the database of commonly misidentified cell lines maintained by ICLAC adn NCBI Biosample

Animals and other organisms

Policy information about studies involving animals; ARRIVE guidelines recommended for reporting animal research

Laboratory animals	NOD.Cg-Prkdcscid Il2rgtm1Wjl/SzJ (NSG) male mice (8–10 weeks old, Jackson Laboratories) were used for all survival surgery studies.<br><br>Mouse monocytes were isolated from C57BL/6J mice (5-8 week old, Jackson Laboratories).
Wild animals	This study did not involve wild animals

Field-collected samples	This study did not involve samples collected from the field
Ethics oversight	All mouse studies were carried out according to a Georgia Tech IACUC-approved animal protocol

Note that full information on the approval of the study protocol must also be provided in the manuscript.

Flow Cytometry

Plots

Confirm that:

- [x] The axis labels state the marker and fluorochrome used (e.g. CD4-FITC).
- [x] The axis scales are clearly visible. Include numbers along axes only for bottom left plot of group (a 'group' is an analysis of identical markers).
- [ ] All plots are contour plots with outliers or pseudocolor plots.
- [x] A numerical value for number of cells or percentage (with statistics) is provided.

Methodology

Sample preparation	Flow cytometry was performed on MSC that were cultured in TCP coated dishes.
Instrument	FACSAria III flow cytometer (BD Biosciences)
Software	Data was collected using BD FACSDiVa software (BD Biosciences)<br>Flow data was analyzed using FlowJo V10
Cell population abundance	10,000 events were counted per sample of cells
Gating strategy	For hMSC and integrin marker expression, no gating was performed. Stained samples were compared to isotype controls.<br>For the luciferase transfected hMSC flow, the gate was drawn based on the untransfected population.

- [ ] Tick this box to confirm that a figure exemplifying the gating strategy is provided in the Supplementary Information.
